# Design and Synthesis of Non-Peptide Mimetics Mapping the Immunodominant Myelin Basic Protein (MBP_83–96_) Epitope to Function as T-Cell Receptor Antagonists

**DOI:** 10.3390/ijms18061215

**Published:** 2017-06-08

**Authors:** Mary-Patricia Yannakakis, Carmen Simal, Haralambos Tzoupis, Maria Rodi, Narges Dargahi, Monica Prakash, Athanasia Mouzaki, James A. Platts, Vasso Apostolopoulos, Theodore V. Tselios

**Affiliations:** 1 Department of Chemistry, University of Patras, 26504 Rion Patras, Greece; yannakakism@gmail.com (M.-P.Y.); carmen.simal@gmail.com (C.S.); haralambostz@gmail.com (H.T.); 2 School of Chemistry, Cardiff University, Park Place, Cardiff CF103AT, Wales, UK; platts@cardiff.ac.uk; 3 Laboratory of Immunohematology, Division of Hematology, Department of Internal Medicine, Medical School, University of Patras, Rion, 26500 Patras, Greece; marodi_biol@yahoo.gr (M.R.); mouzaki@upatras.gr (A.M.); 4 Centre for Chronic Disease, College of Health and Biomedicine, Victoria University, St. Albans, VIC 3021, Australia; Narges.dargahi@live.vu.edu.au (N.D.); monica.prakash@vu.edu.au (M.P.)

**Keywords:** multiple sclerosis, trimolecular complex, rational drug design, non-peptide mimetics, molecular modeling, cell proliferation, T cell antagonism

## Abstract

Encephalitogenic T cells are heavily implicated in the pathogenesis of multiple sclerosis (MS), an autoimmune demyelinating disease of the central nervous system. Their stimulation is triggered by the formation of a trimolecular complex between the human leukocyte antigen (HLA), an immunodominant myelin basic protein (MBP) epitope, and the T cell receptor (TCR). We detail herein our studies directed towards the rational design and synthesis of non-peptide mimetic molecules, based on the immunodominant MBP_83–96_ epitope that is recognized by the TCR in complex with HLA. We focused our attention on the inhibition of the trimolecular complex formation and consequently the inhibition of proliferation of activated T cells. A structure-based pharmacophore model was generated, in view of the interactions between the TCR and the HLA-MBP_83–96_ complex. As a result, new candidate molecules were designed based on lead compounds obtained through the ZINC database. Moreover, semi-empirical and density functional theory methods were applied for the prediction of the binding energy between the proposed non-peptide mimetics and the TCR. We synthesized six molecules that were further evaluated in vitro as TCR antagonists. Analogues **15** and **16** were able to inhibit to some extent the stimulation of T cells by the immunodominant MBP_83–99_ peptide from immunized mice. Inhibition was followed to a lesser degree by analogues **17** and **18** and then by analogue **19**. These studies show that lead compounds **15** and **16** may be used for immunotherapy against MS.

## 1. Introduction

Multiple sclerosis (MS) is an immunologically controlled, inflammatory, demyelinating disease, described as the destruction of the myelin sheath of the central nervous system, which can lead to paralysis [1,2]. Although evidence suggests the important role of B-cells (auto-antibodies), T helper (Th)-17 cells, and CD8^+^ T cells in disease pathogenesis [3], it is well regarded that CD4^+^ Th1 cells contribute to initiation and progression of disease. Indeed, CD4^+^ T cells have been identified in patients with MS to react to self-peptide epitopes within the myelin sheath, including that of myelin basic protein (MBP), proteolipid protein, myelin oligodendrocyte glycoprotein, and myelin associated glycoprotein [4,5]. In the context of MS, encephalitogenic T cells are activated through the formation of a trimolecular complex between the T cell receptor (TCR), a short 14–18 amino acid myelin peptide (epitope), and the major histocompatibility complex (MHC) class II. In fact, the MHC class II, human leukocyte antigen (HLA) locus is the most closely correlated genetic locus to the development of MS, in particular HLA-DR1, HLA-DR2, and HLA-DR4 [6,7,8]. In humans, the MHC class II (HLA) consists of dimers (the α chain and the β chain) [9,10], which present short antigenic peptide epitopes to CD4^+^ Th cells, resulting in the formation of the trimolecular complex (HLA-peptide-TCR). The TCR is also composed of two different polypeptide chains (α and β chains) that consist of variable domains (complementarity determining regions; CDRs). CDRs are implicated in the recognition of the TCR to HLA-peptide complex, and their structural diversity plays a crucial role in the recognition of the different antigens presented to T cells by antigen presenting cells [11,12]. In fact, there are more than 2.5 × 10^7^ unique TCR (CDRs) structures in humans [12]. In addition, the rigorous positive and negative selection process of T cells in the thymus does not prevent auto-reactive T cells from escaping thymic deletion [13,14,15], thus initiating the development of autoimmune disorders such as MS.

In patients with MS, T cell responses are primarily associated with recognition of the 81–105 region of MBP (QDENPVVHFFKNIVTPRTPPPSQGK) [16], with the MBP_83–99_ (ENPVVHFFKNIVTPRTP) peptide epitope displaying the strongest binding to HLA-DR2 [17,18], MBP_83–96_ being the minimal recognized epitope. T cell recognition of MBP_83–96_ has also been shown in healthy individuals, albeit at relatively low precursor frequencies [19]. Hence, the immunodominant MBP_83–96_ epitope plays an important role at inducing CD4^+^ T cells, which contribute to the demyelination process, and it is therefore considered one of the main targets for developing molecular therapeutics [20,21]. The primary binding residues of MBP_83–96_ to HLA-DR2 are via hydrophobic V^87^ and F^90^, which anchor the peptide into pockets P1 and P4, respectively, as noted in the HLA-DR2-peptide-TCR crystal structure [22]; albeit at a low resolution of 3.5 A, this structure served as the basis of all future studies of MBP peptides interacting with HLA-DR2. Additionally, other crystal structures reported in the RCSB databank [23,24] that address the role of MBP immunodominant epitopes in MS induction contain the same TCR sequence. Furthermore, it was noted that a second step in the T cell activation process involves the recognition of His^88^ and Phe^89^, which are placed in pockets P2 and P3 of the TCR [22], with secondary binding residues being Val^86^ and Lys^91^, which are oriented in pockets P-1 and P5 of the TCR [22]. Thus, a detailed analysis of the interactions between HLA-MBP_83–96_-TCR complexes would lead to valuable information towards rational design of non-peptide mimetics with inhibitory activity. Indeed, a number of studies have shown that using antagonist peptides (1–2 amino acid mutations to TCR contact residues), or altered peptide ligands, can effectively modulate T cell responses and switch from pro- to anti-inflammatory responses [25,26,27,28,29,30,31,32,33,34,35]. In addition, using a computational structure-based approach, non-peptide mimetics of small organic compounds that were able to bind to MHC class II and block the presentation of MBP_152–185_ to auto-reactive T cells were identified [36].

The principal goal of this study was the rational design of non-peptide mimetic molecules that could bind to the TCR with increased affinity and not to the MHC–peptide complex. Such potential inhibitors would prevent the formation of the trimolecular complex and consequently the stimulation of T cells. To this end, robust computational techniques, such as molecular docking, pharmacophore modeling, and molecular dynamics, were utilized for the design of novel TCR inhibitors. The application of pharmacophore modeling in the trimolecular complex (HLA-MBP_83–96_-TCR) allows the differentiation between the different contributions (e.g., electrostatic and van der Waals interactions, hydrogen donors and acceptors) involved in the epitope recognition process. By analyzing the variations in these aspects, it is possible to search through diverse chemical databases and filter the results for the identification of potential lead TCR antagonists (hits). Furthermore, molecular docking methodologies can be implemented in order to identify and isolate common substructures of the top ranking hits. Subsequently, the analogue with the best docking score (lead molecule) and preferable structural orientation over the TCR is selected for further optimization and this optimized structure then opts for increased interactions with the TCR. Molecular dynamics (MD) simulations and molecular orbital calculations were carried out in the optimized hits in order to evaluate their binding to the TCR. Finally, the proposed analogues were synthesized to evaluate their biological activity against MBP_83–99_ primed mouse T cells and to human peripheral blood T cells.

## 2. Results and Discussion

### 2.1. Pharmacophore Modeling and Virtual Screening

**Figure 1:**
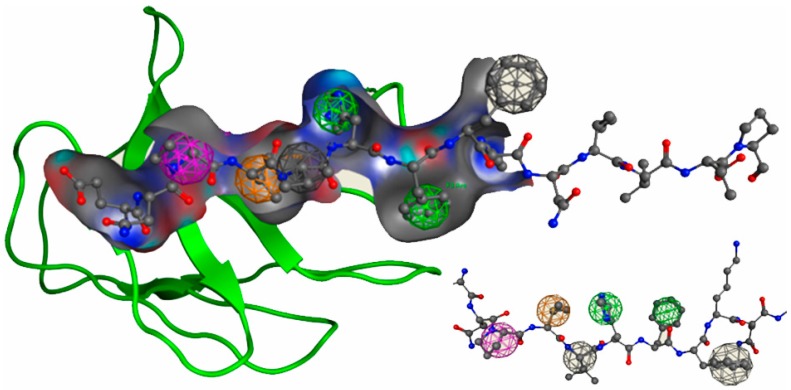
The proposed pharmacophore model, based on the myelin basic protein MBP_83–96_ epitope, with the relevant features depicted as spheres (Aro: green; Cat: magenta; Hyd: orange; V: gray). Only the binding cavity of the T cell receptor (TCR) is presented in the figure as surface and ribbons. The residues of the MBP_83–96_ are depicted as sticks.

In computational drug discovery, screening of large databases with chemical property information obtained from relatively small data is essential. The combination of results from structure- and ligand-based pharmacophore models allows a thorough search in order to discover potential antagonists. The proposed pharmacophore model (Figure 1) is based on features such as an aromatic ring (Aro, green), a hydrogen bond (HB), cation and donor (Cat, magenta), hydrophobic groups (Hyd, orange), and volume exclusion (V, gray). The detailed parameters utilized for the construction of the model are described in the Materials and Methods section. The key features are based on residues His^88^ and Phe^89^ (Aro, Figure 1, green sphere), Val^86^ (Hyd, Figure 1 orange sphere), and Pro^85^ (Cat, Figure 1, magenta sphere). The grey spheres in Figure 1 represent residues with bulky side chains, such as Val^87^ and Phe^90^, that do not interact with the TCR. These residues are employed to define the Volume Exclusion (V) feature of the pharmacophore model. This information is important for excluding residues that interact with the HLA receptor and consequently are not involved in key interactions with the TCR.

The next step was the implementation of the pharmacophore model for the virtual screening of chemical databases. As described in the Materials and Methods section; the ZINC database was employed in the virtual screening process. The combinatorial information yielded from the pharmacophore model was employed as the starting point of our search. The examination of compounds in databases yielded a total of 340 potential inhibitors (hits). A subsequent visual analysis revealed 13 molecules (compounds **1**–**13**, Table 1) with binding conformations that closely resembled the positioning of the MBP_83–96_ epitope inside the TCR binding cavity (Table 1 and Table S1).

### 2.2. Lead Optimization and Molecular Docking Calculations

All the selected molecules were visualized in MOE2010, while their structural orientation and binding with the TCR were assessed. Each of the potential hits was subjected to molecular docking calculations, and the results are presented in Table 1. The analysis of the docking experiments showed that, of the 13 compounds obtained from the pharmacophore screen, compound **10** presented with the highest docking score (−21.56 kcal/mol) inside the TCR binding cavity, while the lowest docking score was reported for compound **5** (−10.32 kcal/mol). This suggests that compound **10** may be considered the best candidate for lead optimization. The formation of only 2 hydrogen bonds with residues AspA92 and GlyA96 of the TCR is noted along with the existence of a π-stacking interaction between the aromatic rings of the compound and the side chain of TyrA98 in the TCR. Despite the favorable interactions between analogue **10** and the TCR, the bulky nature of the lead compound prevents the better positioning of the molecule inside the binding cavity.

**Figure 2:**
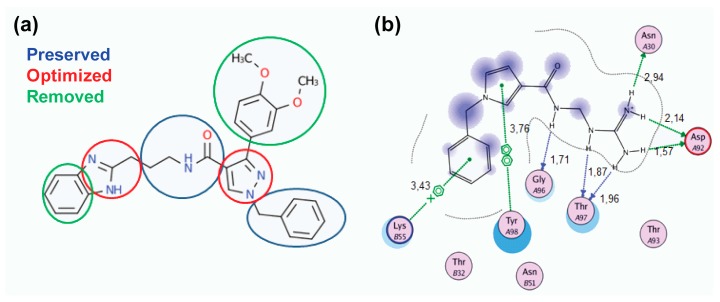
(**a**) Optimization process for the lead compound **10**. The preserved groups are presented in blue, while the optimized groups are shown in red and the removed ones in green; (**b**) best docking pose of compound **14** inside the TCR binding site, showing the different interactions. Green arrow: Hydrogen Bond (HB) formed with the side chain of the residue; Blue arrow: HB formed with the backbone; Blue shade: Solvent accessible surface area (SASA) of the ligand; Turquoise halo: SASA of the receptor residues; Green dotted lines: π-π interactions between two phenyl rings or interaction of a cation (+) with a π system (phenyl ring).

The optimization process for target compound **10** included the removal and addition of functional groups in order to improve the placement of the molecule inside the selected TCR pockets and subsequently increase the interactions (Figure 2a). As depicted in Figure 2a, the substituted aromatic ring was removed to decrease the bulky nature of the potential inhibitor. The benzimidazole was replaced by a guanidino group (Figure 2a) to enhance the hydrogen bonding potential of the designed inhibitor. This preliminary study led to the identification of compound **14** as drug-target (Table 1 and Figure 2b). The next step was the setup of a molecular docking simulation for compound **14** in the TCR. The results of the docking experiments show that the alterations in compound **14** increase its binding affinity inside the TCR compared to the lead compound **10** (−23.76 to −21.56 kcal/mol, Table 1). The ligand pose with the best docking score for compound **14** (Figure 2b) presented the formation of six hydrogen bonds with residues of the TCR. In addition to the hydrogen bond interactions with amino acids AspA92 and GlyA96, the optimized compound further interacts with residues AsnA30 and ThrA97 (Figure 2b). The improved interaction, via the increased number of hydrogen bonds, may further explain the better binding affinity of compound **14**, due to the more favorable positioning inside the binding cavity of the receptor. As expected the π-stacking interaction with TyrA98 in the TCR is retained in the new optimized compound, further enhancing its binding.

As stated in the Materials and Methods section, the filtering process of the pharmacophore search was based on Lipinski’s rule of five. Properties such as size (molecular weight, MW), hydrophobicity content (logP), and Total Polar Surface Area (TPSA) were recorded for the potential candidates (Table S1). The lead compound (compound **10**) was selected due to its high binding affinity (Table 2) and its better positioning inside the TCR binding cavity. The optimization process that led to the design of compound **14** aimed to enhance the binding affinity as well as to improve its positioning deeper within the TCR. Additionally, the modifications in the lead compound were intended to reduce its hydrophobic content (logP) and increase the polar surface area of the proposed inhibitor (Table 2). The smaller size of optimized compound **14** (MW = 272.33, Table 2), showed a notable decrease in its hydrophobic content (−0.84 from 5.25 of compound **10**, Table 2) and an increase in its TPSA (Table 2). Both of these chemical properties are indicators of compound's membrane/cell permeability. Compound **14** proved better potential absorption properties than the lead compound, as indicated by the logP and TPSA values.

**Figure 3:**
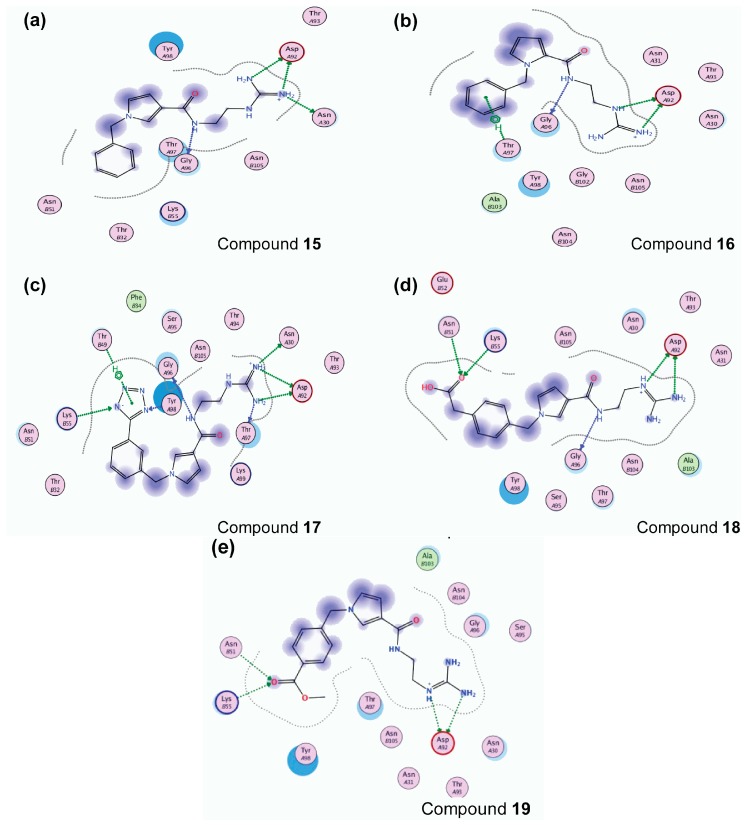
Best docking poses inside the TCR binding site, showing the different interactions for compounds: (**a**) **15**; (**b**) **16**; (**c**) **17**, (**d**) **18**; (**e**) **19**. Green arrow: Hydrogen Bond (HB) formed with the side chain of the residue; Blue arrow: HB formed with the backbone; Blue shade: Solvent accessible surface area (SASA) of the ligand; Turquoise halo: SASA of the receptor residues; Green dotted lines: interaction of C-H with a π system (phenyl ring).

Based on the calculated properties of the compounds **10** and **14** (logP and TPSA, Table 2), we aimed to further optimize analogue **14** through small changes in the compound’s backbone to explore whether an additional increase in binding affinity is possible. Thus, two new target molecules **15** and **16** were obtained (Table 2, Figure 3); the 3–substituted pyrrole ring with an additional methylene group (–CH_2_–) between the amide bond and the guanidino group, compound **15** (Figure 3a), and its 2–substituted pyrrole ring isomer, compound **16** (Figure 3b). The addition of the methylene group aimed to improve the positioning of the guanidino group in the P2 pocket of TCR. As expected, this variation increases the molecular weight but does not affect the hydrophobicity content, and the TPSA values of the two derivatives in comparison to compound **14** (Table 2). Molecular docking simulations were also carried out for analogues **15** and **16** in complex with the TCR, and the results are reported in Table 2 and Figure 3. The reported interactions for compounds **15** and **16** show the retention of the hydrogen bonds with AspA92 and GlyA96 (Figure 3a,b), while the addition of the methylene group prevents the interactions with residues AsnA30 and ThrA97 reported for compound **14** (Figure 2b and Figure 3a,b). The absence of these interactions, compared to compound **14**, may explain the differences observed for the binding affinities of the two derivatives **15** and **16** (Table 2).

The abolition of interactions with residues AsnA30 and ThrA97 for analogue **15** and the subsequent decrease in the binding affinity compared to compound **14** (Table 2) led to the design of derivatives **17**–**19** (Figure 3c–e). The analogues include meta– (compound **17**) and para– (compounds **18** and **19**) substitutions of the aromatic ring in compound **15**. The meta– substitution with the tetrazole group in compound **17** restores the hydrogen bond with ThrA97 (Figure 3c). Furthermore, the tetrazole interacts via the formation of a hydrogen bond with TyrA98 (Figure 3c). The new interactions between the compound and TCR residues are mirrored in the increased docking score of the molecule as reported in Table 2. The para –CH_2_COOH substitute (compound **18**) retains the interactions of analogue **15** with AspA92 and GlyA96, while creating hydrogen bonds with residues AsnB51 and LysB55 (Figure 3d). The amino acids AsnB51 and LysB55 are located in the TCR binding site, opposite to AspA92 and GlyA96, thus enhancing the positioning of derivative **18** in the TCR binding cavity. A similar pattern of interactions inside the TCR cavity is observed for the para– methyl ester substituent (compound **19**, Figure 3e). Again, the methyl ester group allows the compound to be better oriented inside the binding site. The possible advantageous positioning of compounds **18** and **19** is mirrored in their docking scores (−20.70 and −21.32 kcal/mol, respectively, Table 2).

### 2.3. Molecular Dynamics Simulations

Molecular dynamics (MD) simulation experiments were performed on the optimized compounds (**14**–**19**). The best docking poses were utilized as the starting conformations in the different MD simulation runs. The conformational changes observed for the TCR are similar in the different MD simulation runs (Figure S1a). This pattern is also observed in the atomic positional fluctuations of the residues of the TCR (Figure S1b). The different amino acids of the receptor show an identical pattern of deviation from their original position in the complexes with different analogues. Furthermore, the conformational analysis of the ligands showed that there are no extensive conformational changes (Figure S1c) during the simulation time. The average RMS value (1.97 Å ± 0.1) for compound **14** presents the greatest deviation from its starting conformation compared to compounds **15** (1.90 Å ± 0.37), **16** (1.72 Å ± 0.20), **17** (1.82 Å ± 0.63), and **18** (1.01 Å ± 0.13). Only analogue **19** presents a higher average RMS value (2.01 Å ± 0.47) to all the other derivatives (Figure S1c). These deviations in the RMS values for the designed analogues reflect very small changes in their conformation during MD simulations.

**Figure 4:**
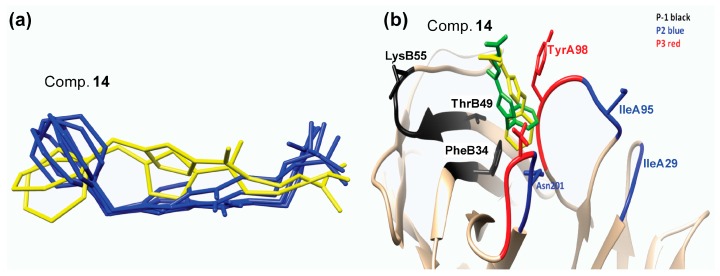
(**a**) Representative conformations of compound **14** inside the TCR as reported by the clustering analysis in the molecular dynamics (MD) simulations and (**b**) the positioning of analogue **14** representative conformations (yellow and green) inside the TCR binding pockets.

**Figure 5:**
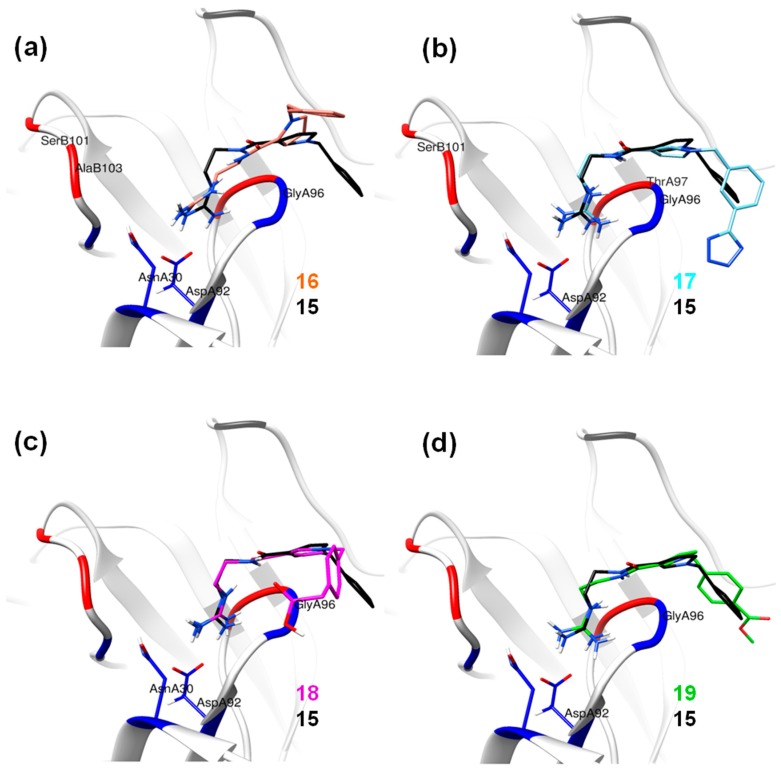
Conformations of compounds **15**–**19** inside the TCR binding pockets. The common backbone features have been implemented for the superimposition of the derivatives on analogue **15** (black); (**a**) with **16** (salmon); (**b**) with **17** (cyan); (**c**) with **18** (magenta); (**d**) with **19** (green).

The clustering analysis for the different MD simulations showed that compound **14** presents two dominant conformational groups throughout the simulation (Figure 4a, blue and yellow). The difference between the two conformations is in the positioning of the aromatic ring inside the P3 pocket of the TCR (Figure 4b). In one instance, the aromatic ring is facing towards TyrA98 (Figure 4b, green) and in the other it faces away from TyrA98 and towards PheB34 (Figure 4b, yellow). In both cases, though, the docking pose is not retained throughout the MD simulation and the guanidino group is facing away from the binding cavity of the TCR (Figure 4b). The modification of compound **14** in which an additional methylene group (analogues **15** and **16**) is introduced might lead to a better positioning inside the TCR binding cavity. The clustering analysis for the two modified analogues **15** and **16** revealed the presence of only one dominant conformation for both compounds (Figure 5a, black and salmon respectively). The positioning of the two analogues **15** and **16** inside the binding cavity of the TCR is very similar (Figure 5a, black and salmon, respectively). The most pronounced difference between them is the positioning of the aromatic ring. In 2–substituted pyrrole analogue **16**, the aromatic ring during the MD simulations points away from the binding pockets (Figure 5a, salmon). On the other hand, 3–substituted pyrrole analogue **15** adopts a more optimal conformation inside the binding pockets of TCR (Figure 5a, black). While analogues **14** and **16** have a portion of their structure pointing away from the TCR receptor (Figure 4b and Figure 5a), the addition of the methylene group in compound **15** allows for the conformation of the molecule to create a bent, thus optimizing the orientation inside pockets P–1, P2, and P3 of the TCR (Figure 5a, black).

As previously mentioned, the best possible positioning of compound **15** inside the binding pocket observed in the docking experiments led to the design of derivatives **17**–**19**. The clustering analysis of the particular simulations confirmed the results obtained from the RMS analysis (Figure S1c). Likewise, with compounds **15** and **16**, the derivatives **17**–**19** present only one dominant conformation throughout the MD simulations. The structural similarities of analogue **15** with compounds **17**–**19** led to the supposition that the derivatives would adopt a similar positioning inside the TCR. The superimposition of the representative conformations with that of compound **15** (Figure 5) confirmed the above supposition. Compounds **17** and **18** present identical positioning inside pockets P2 and P3 with that of compound **15** (Figure 5b,c), suggesting that the guanidino group firmly anchors the analogues inside the receptor. At the opposite end of the derivatives though the substitutions with the tetrazole (compound **17**) and the –CH_2_COOH (compound **18**) groups do not greatly improve the conformational positioning of the designed analogues in the binding cavity. Additionally, the –CH_2_COOH substituent in compound **18** orients the aromatic ring of the derivative away from the pockets of TCR (Figure 5c). On the other hand, the positioning of compound **19**, which has a para– methyl ester substitution, in the binding site of the receptor closely resembles that of analogue **15** (Figure 5d, green). The substitution seems to position the analogue inside the TCR between pockets P3 and P–1 in an even better way (Figure 5d, red and black).

#### Hydrogen Bond Interactions

Analysis of the hydrogen bond interactions for all compounds (**14**–**19**) was performed during the MD simulations. The results are outlined in Table 3 and compared with the interactions reported from the molecular docking experiments. The changes in the orientation of the molecules inside the TCR are mirrored in the observed differences of the interactions for each molecule. As mentioned above, compound **14** creates hydrogen bonds with residues in pockets P2 and P3 (Figure 2b and Table 3) with the guanidino group anchoring the compound in pocket P2 (AsnA30) and pocket P3 (ThrA97). During the MD simulation time, these interactions are not retained, and the terminal nitrogens of the guanidino group do not create stable interactions with the TCR. Instead the only interactions are those with residues of P2 pocket of TCR (AsnB104 and GluB106). The same pattern is observed for compound **16**, where the interactions with residues AspA92 and GlyA96 (P2 pocket of TCR) are not retained during the MD simulations. Instead, analogue **16** is involved in hydrogen bonding interactions with residues TyrA98 and AlaB103, both in the P3 pocket of the receptor (Table 3).

In contrast to the previous two analogues, compound **15** retains the hydrogen interactions reported in the molecular docking experiments (Figure 3a, Table 3). The hydrogen bonds with residues AsnA30 and GlyA96 in the P2 pocket of the TCR are conserved, while the orientation of the molecule inside the cavity allows for interaction with ThrA97 in the P3 pocket (Table 3). Furthermore, the anchoring of the compound **15** inside the two pockets (P2 and P3, Figure 5a), in combination with the bent conformation of the molecule, allows better positioning of the aromatic ring inside the P–1 pocket (Figure 5a). This may lead to increased interactions between the potential inhibitor and the receptor. Similarly to compound **15**, the three derivatives (**17**–**19**) present comparative interaction patterns (Table 3). The guanidino group of these analogues retains the interaction with AspA30 and GlyA96 in the P2 pocket of the TCR observed for compound **15** (Table 3), while there are small changes in the interaction patterns with the neighboring amino acids. Compound **17** further interacts with ThrA97 and SerB101 in the P2 pocket, while compound **18** further interacts with AsnA30 in the P3 pocket and TyrA98 in the P2 pocket of the receptor. Finally, the very similar positioning of compounds **15** and **19** (Figure 5d) points to the conservation of the interactions between the two designed analogues (Table 3). The only difference is the hydrogen bond of compound **19** with ThrA93 instead of GlyA96 in the P2 pocket of the receptor.

### 2.4. Chemistry

**Scheme 1:**
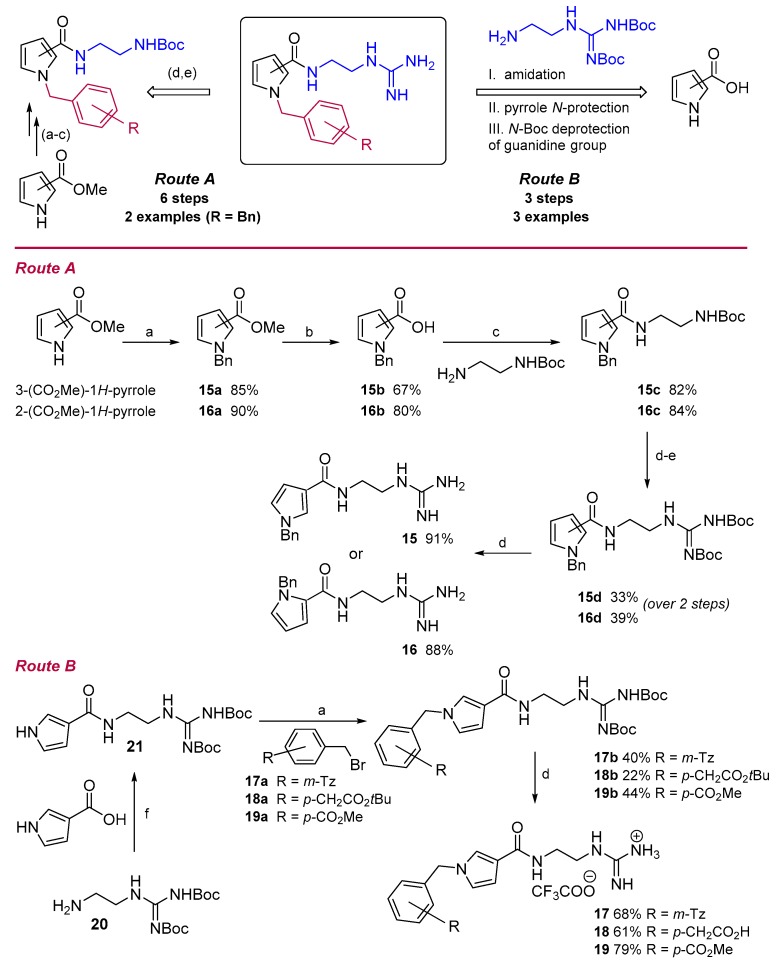
Synthesis of 2-/3-substituted pyrrole analogues **15**–**19**. Reagents and conditions: Route A: (**a**) BnBr, NaH, DMF; (**b**) i. KOH 30%, MeOH–H_2_O, reflux; ii. 6 M HCl; (**c**) DCC, DMAP, DCM; (**d**) TFA, DCM; (**e**) BocNHC (S) NHBoc, NIS, DIPEA, MeOH–DCM; then (**d**) TFA, DCM. Route B: (**a**) BnBr, NaH, DMF; (**f**) DCC/HOBt, DIPEA, DMF–DCM; (**d**) TFA, DCM.

Initial studies on the synthesis of pyrrole-based TCR antagonists provided candidates **15** and **16** via a six-step synthetic procedure with a total yield of 14% and 21%, respectively (Route A, Scheme 1). *N*-alkylation of commercially available 3- or 2-methyl pyrrolecarboxylates, with benzyl bromide in the presence of sodium hydride, afforded the 3-/2-substituted *N*-benzylpyrroles **15a**/**16a** [37]. Subsequent hydrolysis of the methyl ester, followed by standard procedure for DCC/DMAP amide coupling with *N*-Boc-ethylenediamine, gave the corresponding pyrrole carboxamides **15c**/**16c**. *N*-Boc-deprotection with TFA followed by *N*-iodosuccinimide-mediated guanylation reaction [38] with di-Boc-thiourea furnished the di-Boc-guanidino derivatives **15d**/**16d**, which allowed final molecules **15**/**16**, after Boc cleavage.

Upon further investigations, a rapid and simple three-step protocol (Route B, Scheme 1) was developed to expand the scope and utility of this synthetic methodology and readily prepare diverse pyrrole analogues. Thus, the guanidine moiety **20** [39] was first synthesized and then reacted with pyrrole-3-carboxylic acid to provide a common structural core **21**, after amidation reaction. Subsequent pyrrole-*N*-protection [37] with primary alkyl bromides **17a**–**19a**, followed by removal of the Boc-groups, produced target compounds **17**–**19** in a shorter sequence and an 11–27% overall yield.

### 2.5. Molecular Orbital Calculations

From the three analogues (**14**–**16**) reported in this study, compound **15** presents a high docking score (−18.13 kcal/mol) coupled with a preferred orientation inside the binding cavity of the TCR (Figure 5a). This, in combination with the compound’s favorable pharmacokinetic properties (TPSA and logP, Table 2), inspired us to explore the analogue **15**/TCR complex by employing molecular orbital methods.

#### 2.5.1. Semi-Empirical Simulation Method

In order to better estimate the interaction energy of the system, a number of different approaches were employed. The results (Table S2) show that the PM7 (parameterization method 7) [40] approach best reproduces the density functional theory (DFT) calculations for the selected residues. All other semi-empirical (SE) methods tested present considerable errors compared to PM7 despite the inclusion of dispersion correction. Based on these observations, the PM7 method was used as the most appropriate for further calculations on the entire receptor–ligand complex (Table S2). Two protocols were utilized for our calculations. In the first one, the ligand along with the same residues used in the DFT calculations was preferred. The interaction energy of the particular system was calculated to −24.09 (kcal/mol). The larger value compared to the DFT calculations (−31.63/−42.85 kcal/mol) may be attributed to the level of accuracy for the SE methodologies and the treatment of the electron density of the various atoms in the system.

**Figure 6:**
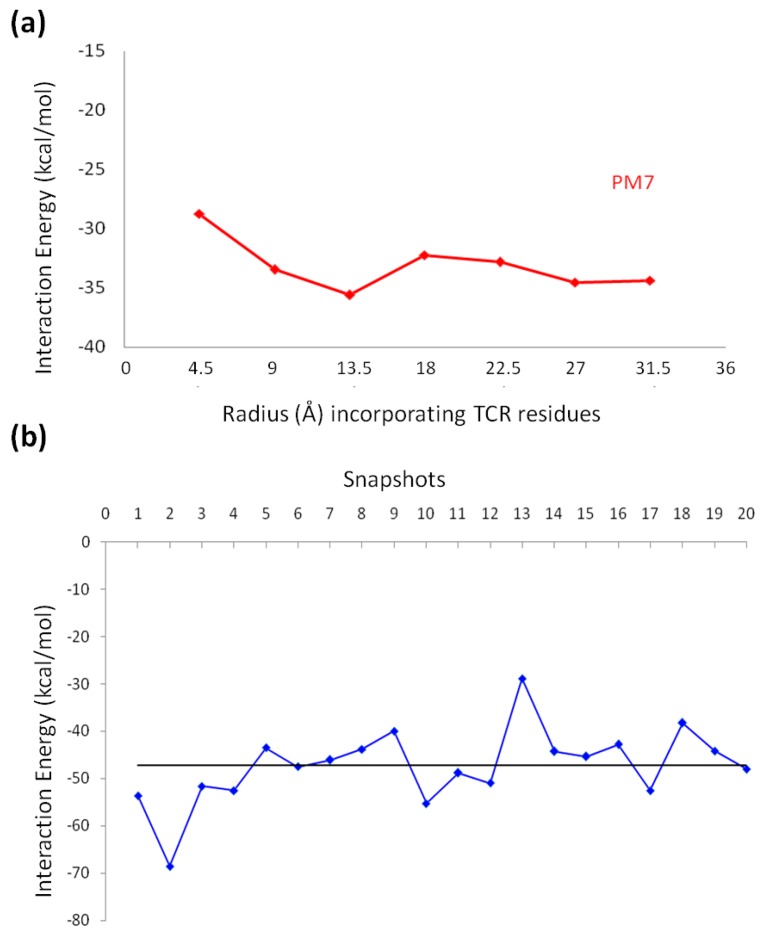
(**a**) Interaction energies for derivative **15** and the residues of the binding cavity, extended by 4.5 Å until the entire TCR is included, calculated by the PM7 method (red) in solvent and (**b**) graphical representation of the interaction energy calculated via the PM7 method for the different MD snapshots.

The second approach employed in our calculations involved the ligand with the whole receptor. In order to explore the effect of the different TCR residues, amino acids within a cutoff distance of 4.5 Å from the ligand were initially elected. Subsequent rounds of interaction energy calculations followed, by augmenting the selected area per 4.5 Å each time until the entire receptor was included in our calculations (Figure 6a). The interaction energy calculated for the TCR in complex with compound **15** is −34.39 kcal/mol. In order to further study the interaction energy of compound **15**, different snapshots were taken from the MD simulation run (the last 20 ns of the simulation). For each snapshot, the interaction energy was calculated with the PM7 method to monitor the fluctuations in the energy (Figure 6b and Table S3). The mean value over the 20 snapshots for the interaction energy was −47.26 kcal/mol. The low interaction energy calculated from both the best docking pose and the different MD snapshots (Tables S2 and S3) suggests that derivative **15** interacts strongly with the TCR and thus may be competitive with native ligands. The interaction energy calculated for compounds **17**–**19** with the SE methodology are reported in Table S5. The values range between −35.39 and −37.20 kcal/mol, higher than the value reported for analogue **15** (−47.26 kcal/mol).

#### 2.5.2. DFT Calculations

The large size of the TCR (341 amino acids) hinders the use of DFT methodologies on the entire complex [41,42]. Thus, to calculate the interaction energy for the complex, the best docking pose was selected. The selection of the receptor residues (total of nine amino acids) was based on the interactions formed with compound **15** and their distance from it (<3.5 Å). As reported in the Materials and Methods section, Section 3.6, different methodologies were employed, and the results are outlined in Table S2. The analysis of the calculations showed variability depending on the method and the basis set selected. In fact, the methods that include dispersion either explicitly or implicitly (e.g., M06, M06-2X, B97D, BHandH, and B3LYP-D) calculate more negative interaction energies (Table S4) [43,44]. In contrast, the choice of basis set does not have such an extensive impact in the calculation of the interaction energy. Therefore, in order to obtain a more accurate result, the inclusion of dispersion functions was considered in our calculations [45]. Based on this, the interaction energy between compound **15** and the selected residues of the TCR was calculated between −31.63 and −42.85 kcal/mol (Table S4). Compared to the SE methodologies, DFT techniques allow for a more accurate prediction of interaction energy between the ligand and the residues that are directly involved in the binding to TCR. The application of DFT incorporates the effect of all atoms, without any of the approximations (empirical data) applied during the SE calculations.

### 2.6. Biological Assays

#### 2.6.1. Human Peripheral Mononuclear Cells

**Figure 7:**
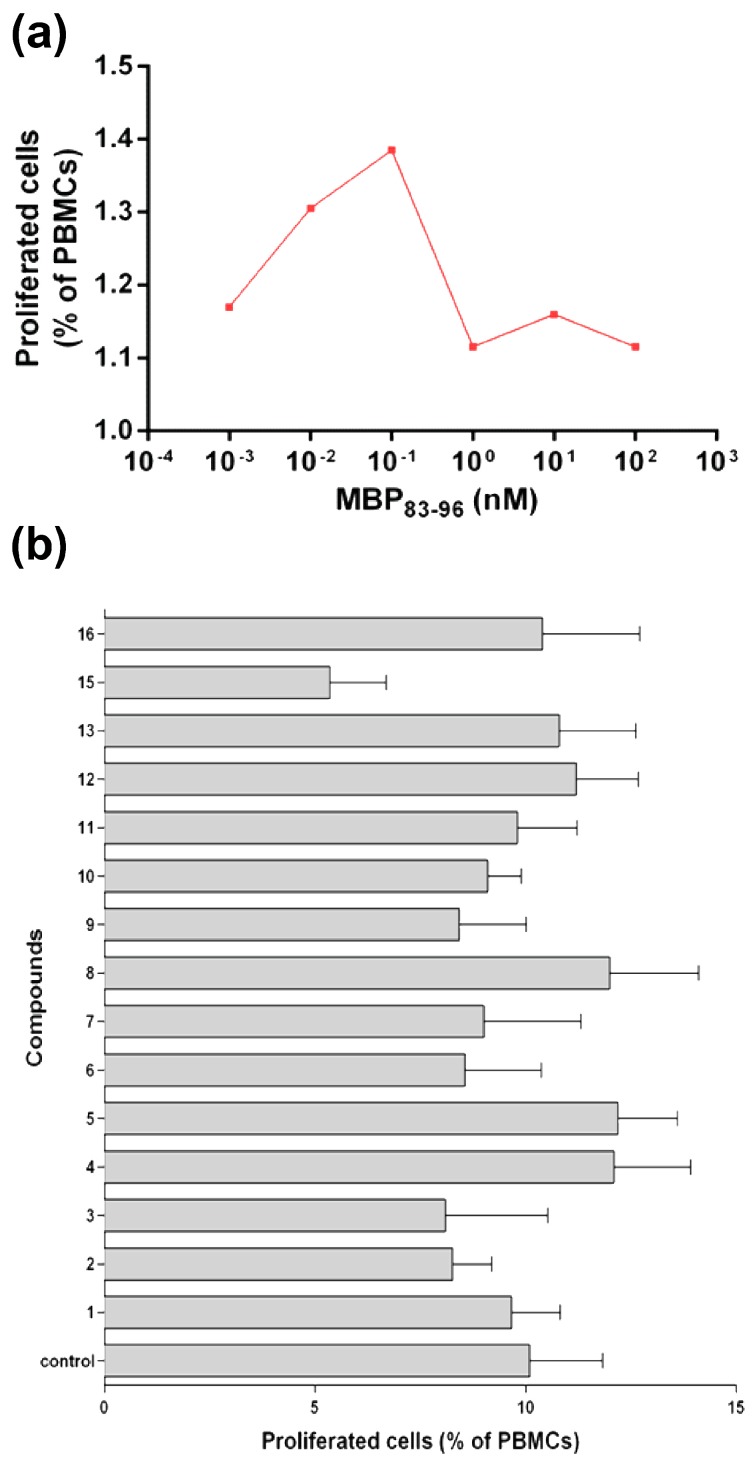
(**a**) T cell proliferation in the presence of MBP_83–96_. Peripheral blood mononuclear cells (PBMC) were cultured with several concentrations of the MBP_83–96_ peptide for 3 days; cell proliferation was measured by flow cytometry. Data are shown as median of triplicate measurements. (**b**) Proliferation of PBMC in the presence of 0.1 nM MBP_83–96_ and 0.1 mM of each of the 15 analogues/point, in triplicate. Control: MBP_83–96_ peptide alone. Data are shown as mean ± standard error of the mean.

Blood samples were drawn from two healthy subjects for biological assays and contained: 1st person: 2.84 × 10^3^ lymphocytes/μL of blood (42.9% of total leukocytes) and 410 monocytes/μL of blood (6.2% of total leukocytes); 2nd person: 1.83 × 10^3^ lymphocytes/μL of blood (34.6% of total leukocytes) and 330 monocytes/μL of blood (6.2% of total leukocytes). The peripheral blood mononuclear cells (PBMCs) isolated from the blood samples were cultured in the presence of various concentrations of the MBP_83–96_ peptide to estimate the optimal concentration for inducing T-cell proliferation. It is noteworthy that the specific culture conditions used in this work, i.e., allo-peptidic antigens and anti-CD28 antibody, target T-cell responses [46]. T-cell proliferation was measured by flow cytometry. The highest T-cell proliferation was noted at 0.1 nM MBP_83–96_ (Figure 7a). PBMC cultures were then repeated with 0.1 nM MBP_83–96_ and 0.1 mM of each of the fifteen analogues (Figure 7, Table 1: compounds **1**–**13**, **15**, and **16**) per experimental point, in triplicate. The results show that analogue **15** was the most effective TCR antagonist, i.e., it conferred the highest inhibition of T cell proliferation (Figure 7b).

#### 2.6.2. Mouse MBP_83–99_ Specific T Cell Assays

**Figure 8:**
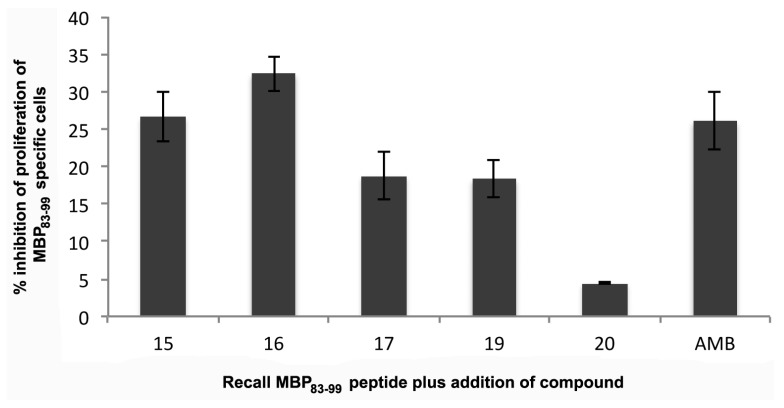
Specific MBP_83–99_ epitope T cell proliferation using MTT as a readout. Mice were immunized three times with reduced mannan conjugated to MBP_83–99_ peptide. Ten days following the last immunization, mice were sacrificed and spleen cells isolated, and MBP_83–99_ peptide was added for 6 days. In addition, compounds **15**–**19** and lead compound **AMB** (lead compound **10**) were added at 100× molar excess. The percent inhibition of T cell proliferation to MBP_83–99_ of each compound is shown. The mean of three individual mice are shown in triplicate wells ± standard error of the mean.

Autoimmune CD4^+^ T cells can be stimulated in mice following immunization with MBP_83–99_ peptide together with *Mycobacterium*, which results in experimental autoimmune encephalomyelitis (EAE), an animal model for MS [47]. Characteristics of EAE are comparable to those of MS in humans where Th1 phenotype (IFN-γ) and Th17 cells contribute to pathogenesis of disease in mice. Similar to MS, EAE susceptibility is dependent on the mouse (MHC class II background) and diverse peptides are immunogenic in different mouse strains. The SJL/J mouse strain (MHC class II H-2^s^ haplotype) is commonly used for EAE, since numerous histopathological, clinical, and immunological features resemble those of human MS [48]. In the SJL/J mouse strain, the peptide MBP_81–100_ has been shown to bind with high affinity to MHC class II, H-2^s^. In fact, the minimum epitope required for binding is MBP_83–99_ [48], similar to human HLA-DR2 binding. Hence, the epitope MBP_83–99_ could be used as an agonist peptide to immunize mice to activate CD4^+^ T cells, as we previously demonstrated [26,30,31,32]. Here, mice were immunized with MBP_83–99_ peptide conjugated to the carrier reduced mannan. Following three immunizations, spleen cells were isolated and mixed with recall peptide MBP_83–99_ for six days in vitro. In addition, compounds **15**–**19** or **AMB** (lead compound **10**) were added at 100× molar excess to each well in order to determine whether T cell proliferation to the recall peptide MBP_83–99_ could be inhibited. The particular compounds (**15**–**19**), due to their increased calculated binding affinity (Table 1) to TCR, were employed in order to assess the potency in mouse MBP_83–99_ specific T cell assays. Compound **15** and **16** showed the greatest % inhibition of MBP_83–99_-specific T cell proliferation, followed by compound **17** and **18**; compound **19** showed the least inhibition, and lead compound AMB was able to inhibit proliferation comparable to that of the other compounds (Figure 8). Compounds **15** and **16** have simpler structures compared to **17**–**19** and **AMB**. It is likely that the reduced activity of **17**–**19** analogues, compared to **15** and **16**, may be due to an inappropriate position of the acidic/esteric group. Even though complete inhibition of T cell proliferation is not noted, compounds **15** and **16**, based on in silico conformational studies, show promise for further optimization studies in order to develop new improved TCR antagonists with improved activity.

## 3. Materials and Methods

### 3.1. Structure Preparation

The X-ray crystallographic coordinates contained in PDB entry 1YMM were obtained from the Protein Data Bank [22]. The particular PDB entry was selected because it contains the main immunodominant epitope MBP_83–96_ involved in MS, as well as a human TCR from a patient with MS. The Molecular Operating Environment (MOE2010) software [49] was utilized for the preparation of the complex. The peptide–TCR complex was isolated, and the residues were protonated accordingly with all hydrogen positions optimized using the AMBER94 force field [50]. All the possible protonation states for the histidine (His) residues were explored and evaluated with the use of the PROpKa [51,52] and AMBER94 tools in MOE2010. The analysis supports the prevalence of neutral His in all cases, in agreement with the results reported by Wucherpfenning et al. [53,54].

### 3.2. Pharmacophore Modeling

The pharmacophore model was designed based on the MBP_83–96_ key residues [55] involved in the binding with the HLA receptor and the TCR. A combination of features from structure- and ligand-based pharmacophore models was utilized in the development of the model presented in this study. According to the crystal structure of the binding cavity of the TCR, an analysis of its chemical features was carried out using the MOE2010 software [49]. The development of the ligand-based pharmacophore model relied on features such as aromaticity (Aro), a hydrogen bond cation (Cat) and donor, and hydrophobic groups (Hyd). The Aro motifs were modeled on the His^88^ and Phe^89^residues of the epitope, the Hyd feature on Val^86^, and the Cat feature on Pro^85^, respectively, all residues that interact with the TCR. The volume exclusion (V) features of the pharmacophore model were developed based on Val^87^ and Phe^90^ that interact with the HLA.

#### Virtual Screening

The pharmacophore hypothesis based on the TCR active site as well as the MBP_83–96_ epitope were utilized to scan 500,000 compounds from the ZINC database [56]. The compounds were filtered according to Lipinski’s rule of five [57] and their commercial availability. Finally, the molecules were sorted based on their fitness to the selected hypothesis.

### 3.3. Molecular Docking

Molecular docking simulations were performed on the TCR using MOE2010 [49]. The ligand, as well as the TCR residues in a radius of 4.5 Å surrounding the ligand, was considered flexible. The definition of the TCR binding site was performed manually by selecting the area including the residues involved in the main binding pockets. The ligands were allowed to move freely in the vicinity of the active site. The top 500 poses for each ligand were ranked using the London *ΔG* scoring function [49]. Subsequently, a maximum of 10 poses were retained based on their docking scoring function, and the poses were rescored using the GBVI/WSA (Generalized-Born Volume Integral/Weighted Surface Area) scoring function [58].

### 3.4. Lead Optimization

Thirteen potential inhibitors (hits) were directly purchased for additional in vitro biological evaluation, as TCR antagonists. Based on their properties and binding scores with the TCR, compound **10** was selected as a lead compound for further optimization. Chemical groups were modified to improve the binding properties, such as orientation of the molecule inside the TCR. Additionally, new chemical groups were added to lengthen the carbon chain and optimize the pocket fit.

### 3.5. Molecular Dynamics (MD) Simulation

The construction of the TCR parameters was performed using the AMBER force field ff14SB [59], while the parameters for the organic molecules were constructed using the general Amber force field (GAFF) [60]. The TIP3P water model [61] was utilized for the solvation of the system, and the total charge was neutralized by the addition of Cl^−^ ions. Truncated octahedral periodic boundary conditions were applied to the system with a cutoff distance of 10 Å. The next step involved minimization, followed by the heating of the system, under a constant volume, to 300 K for 100 ps using the Langevin dynamics temperature scaling [62]. This was followed by equilibration for another 100 ps under constant pressure. Both heating and pressure equilibration were carried out using a 10 kcal·mol^−1^·Å^−2^ restraint on the solute. The equilibration step under constant pressure was prolonged for a further 200 ps, after removing all restraints. The MD production run was performed under constant pressure and temperature conditions (NPT ensemble) for 50 ns. The temperature was kept constant with the use of the Langevin thermostat (using a collision frequency of 2 ps^−1^). All bonds involving hydrogen atoms were kept to their equilibrium distance with the SHAKE algorithm (allowing for a 2 fs time step to be used) [63]. The long range electrostatic interactions were calculated with the Particle Mesh Ewald (PME) method [64]. The different systems were subjected to all-atom unrestrained MD simulations in explicit solvent using AMBER12 [65]. The cpptraj module [66] of AMBER12 was implemented for the trajectory analysis (clustering, RMSD, hydrogen bonds).

### 3.6. Chemistry

Reactions involving moisture sensitive reagents were carried out under an argon atmosphere in addition to oven-drying glassware and anhydrous solvents. Room temperature (rt) refers to 20–25 °C. Crude products were purified by flash chromatography on 230–400 mesh silica gel in the solvents system stated. Analytical thin layer chromatography was performed on pre-coated aluminium plates (Merck 60G F254 silica). TLC visualization was performed out with ultraviolet light (254 nm). The yields were calculated in *w*/*w*. ^1^H and ^13^C nuclear magnetic resonance (^1^H NMR, ^13^C NMR) spectra (Figures S2–S14) were acquired on Bruker Avance 400 and Bruker Ascend 600 spectrometer at ambient temperature in the deuterated solvent stated. All chemical shifts are quoted in parts per million (ppm) relative to the internal standard (TMS). All coupling constants, *J*, are quoted in Hz. Multiplicities are indicated by s (singlet), d (doublet), t (triplet), q (quartet), and m (multiplet). The abbreviation Ar is used to denote aromatic, br to denote broad, and app to denote apparent. Mass spectrometry (*m*/*z*) data were acquired on an Electrospray Ionization Platform spectrometer (ESI-MS) by Micromass and a MassLynx NT 2.3 operating system (Waters Corporation, Milford, MA, USA).

#### 3.6.1. General Procedure A: *N*-Alkylation of Pyrroles

To a solution of 1*H*-pyrrole analogue (1.00 equiv) in DMF (5–10 mL/mmol), under argon at 0 °C was added sodium hydride 60% (1.50–2.50 equiv), and the resulting mixture was stirred at the same temperature for 10–20 min. Then, a solution of the corresponding alkyl bromide (1.00–1.50 equiv) in DMF (5–10 mL/mmol) was added dropwise, and the reaction mixture warmed to rt over ~2 h (monitored by TLC). It was quenched with water (20 mL) and extracted with EtOAc (3 × 20 mL). The combined organics were washed with brine (20 mL), dried (Na_2_SO_4_), filtered, and concentrated in vacuo. Purification of the residue by column chromatography on silica gel (using the appropriate mixture of eluents) allowed pyrroles *N*-protected **15a**, **16a**, and **17b**–**19b**.

#### 3.6.2. General Procedure B: Hydrolysis of Methyl Pyrrole-2/3-Carboxylates

To a solution of methyl *N*-benzyl pyrrole 3- or 2-carboxylate **15a** or **16a** (1.00 equiv) in MeOH–H_2_O 3:1 *v*:*v* (15.0 mL/mmol), an aq solution of KOH 30% (15.0 mL/mmol) was added. The resulting reaction mixture was refluxed and monitored by TLC (10% MeOH–DCM) until completion (~2 h). Then, it was allowed to attain rt and acidified pH = l via the addition of 6.0 M HCl (until cloudiness persisted). The white precipitate was filtered off and washed with ice-water to give the crude of **15b** or **16b**, respectively, which was used in the next step without further purification.

#### 3.6.3. General Procedure C: Amidation Reaction

To a solution of the required pyrrole 3- or 2-carboxylic acid, **15a** or **16a** (1.00 equiv) in dichloromethane (DCM) (20.0 mL/mmol), 4-dimethylaminopyridine (DMAP) (20 mol %), *N*-Boc-ethylenediamine (1.00 equiv), and then *N,N*′-dicyclohexylcarbodiimide (DCC) (1.50 equiv) at 0 °C were added. The resulting mixture was warmed to rt and stirred for a further 16 h (monitored by TLC, 10% MeOH–DCM). After completion of the reaction, dicyclohexylurea (DCU) formed was filtered off and washed with DCM (5 mL) at 0 °C. The organic layer was quenched with 0.1 M HCl (20 mL) and extracted with EtOAc (3 × 20 mL). The combined organics were washed with brine (20 mL), dried (Na_2_SO_4_), filtered, and concentrated in vacuo. Purification of the residue by column chromatography on silica gel (using the appropriate mixture of eluents) allowed pyrrole 3-/2-carboxamides **15c** or **16c**.

#### 3.6.4. General Procedure D: Removal of the Boc-Group

The corresponding *N*-Boc analogue (1.00 equiv) was dissolved in trifluoroacetic acid (TFA)–DCM 95:5 *v*/*v* DCM (20–30 mL/mmol) (and added triethylsilane (TES, 1.00 equiv) if required). The reaction mixture was stirred at rt, and the progress was monitored by TLC (10% MeOH–DCM) until complete consumption of the starting material.

#### 3.6.5. General Procedure E: Guanylation Reaction

The amine salt **15c’** or **16c’** (as crude derived from *N*-Boc deprotection of **15c** or **16c**) was dissolved in a mixture of MeOH–DCM 4:1 *v*:*v* (20.0 mL/mmol), under argon. Then, *N*,*N*′-di-(*tert*-butoxycarbonyl)thiourea (1.50 equiv), *N*,*N*-diisopropylethylamine (DIPEA) (4.00 equiv) and *N*-iodosuccinimide (1.50 equiv) in one portion were added at rt. The reaction mixture was stirred at rt under argon, and monitored by thin layer chromatography (TLC) (20% MeOH–DCM) until completion (~24 h). It was next quenched with an aq solution of 1 M sodium thiosulfate solution (20 mL), and the resulting solution was then diluted in water (20 mL) and extracted with EtOAc (3 × 20 mL). The combined organic layer was washed with brine (20 mL), dried (Na_2_SO_4_), filtered, and concentrated in vacuo. Purification of the residue by column chromatography on silica gel (using the appropriate mixture of eluents) allowed the corresponding di-Boc-guanidino derivatives **15d** or **16d**.

#### 3.6.6. Synthesis of Methyl 1-Benzyl-1*H*-Pyrrole-3-Carboxylate **15a** [67]

From methyl 1*H*-pyrrole-3-carboxylate (98.4 mg, 0.79 mmol) and NaH 60% (38.0 mg, 1.58 mmol) in dimethylformamide (DMF) (4.0 mL), and a solution of benzyl bromide (0.14 mL, 1.18 mmol) in DMF (6.0 mL), following the general procedure A (2 h) and after chromatographic purification (DCM), **15a** (144 mg, 85%) was obtained as a clear gum. Data for **15a**: ^1^Η NMR (400 MHz, CDCl_3_) δ 7.31–7.37 (m, 4H, Ph, Ar), 7.13–7.15 (m, 2H, Ph), 6.60–6.63 (m, 2H, Ar), 5.06 (s, 2H, CH_2_Ph), 3.79 (s, 3H, OMe); ESI-MS *m/z* found for C_13_H_13_NO_2_: 216.32 [M + H]^+^; RP-HPLC gradient separation from 30% to 100% acetonitrile at 30 min, flow rate: 1 mL/min*, t_R_* = 10.8 min.

#### 3.6.7. Synthesis of 1-Benzyl-1*H*-Pyrrole-3-Carboxylic Acid **15b** [68]

From methyl 1-benzyl-1*H*-pyrrole-3-carboxylate **15a** (144 mg, 0.67 mmol) in MeOH–H_2_O (10.0 mL) and an aq solution of KOH 30% (10.0 mL), following the general procedure B (2 h) and after precipitation, the crude of **15b** (90.0 mg, 67%) was used in the next step without further purification. Data for **15b**: proton nuclear magnetic resonance (^1^Η NMR) (400 MHz, CDCl_3_) δ 7.29–7.40 (m, 4H, Ph, Ar), 7.15 (d, 2H, *J* = 7.2 Hz, Ph), 6.63-6.66 (m, 2H, Ar), 5.07 (s, 2H, CH_2_Ph); electrospray ionization mass spectrometry (ESI-MS) *m/z* found for C_12_H_11_NO_2_: 425.41 [2M + Na]^+^, 202.25 [M + H]^+^; RP-HPLC gradient separation from 5% to 100% acetonitrile at 30 min, flow rate: 1 mL/min, *t_R_* = 14.2 min.

#### 3.6.8. Synthesis of 1-Benzyl-1*H*-*N*-[2-(Tert-Butoxycarbonyl)Aminoethyl]Pyrrole-3-Carboxamide **15c**

From 1-benzyl-1*H*-pyrrole-3-carboxylic acid **15b** (90.0 mg, 0.45 mmol) in DCM (9.0 mL), DMAP (10.9 mg, 0.09 mmol), *N*-Boc-ethylenediamine (0.07 mL, 0.45 mmol), and then DCC (138 mg, 0.67 mmol), following the general procedure C (16 h) and after chromatographic purification (20% MeOH–DCM), **15c** (126 mg, 82%) was obtained as a clear gum. Data for **15c**: ^1^Η NMR (400 MHz, CDCl_3_) δ 7.29–7.35 (m, 3H, Ph), 7.25–7.26 (m, 1H, Ar), 7.12–7.14 (m, 2H, Ph), 6.62 (app t, 1H, *J* = 2.4 Hz, Ar), 6.48 (br s, 1H, NH), 6.41 (br s, 1H, Ar), 5.04 (s, 2H, CH_2_Ph), 4.98 (br s, 1H, NH), 3.51–3.45 (m, 2H, CH_2_), 3.32–3.35 (m, 2H, CH_2_), 1.41 (s, 9H, 3× CH_3_*t*-Bu); ESI-MS *m/z* found for C_19_H_25_N_3_O_3_: 344.33 [M + H]^+^, 288.32 [(M–Ph) + Na]^+^; reversed phase high-performance liquid chromatography (RP-HPLC) gradient separation from 5% to 100% acetonitrile at 30 min, flow rate: 1 mL/min*, t*_R_ = 17.0 min.

#### 3.6.9. Synthesis of 1-Benzyl-1*H*-*N*-[2-(2,3-Di-Tert-Butoxycarbonyl)Guanidinoethyl]Pyrrole-3-Carboxamide **15d**

From *N*-Boc analogue **15c** (120 mg, 0.35 mmol) in TFA–DCM 95:5 (7.0 mL), following the general procedure D (1 h), the crude of 2-(1-benzyl-1*H*-pyrrole-3-carboxamido)ethanaminium 2,2,2-trifluoroacetate **15c’** was dissolved in MeOH–DCM (7.0 mL). Then, *N*,*N*′-di-(*tert*-butoxycarbonyl)thiourea (145 mg, 0.52 mmol), DIPEA (0.24 mL, 1.40 mmol, 4.00 equiv), and *N*-iodosuccinimide (118 mg, 0.52 mmol), following the general procedure E (24 h) and after chromatographic purification (20% MeOH–DCM), **15d** (56.6 mg, 33%) was obtained as a clear gum. Partial data for **15c’**: ESI-MS *m/z* found for C_14_H_17_N_3_O: 244 [M]^+^; RP-HPLC gradient separation from 5% to 100% acetonitrile at 30 min, flow rate: 1 mL/min*, t*_R_ = 10.3 min. Data for **15d**: ^1^Η NMR (400 MHz, CDCl_3_) δ 11.50 (s, 1H, NH), 8.71 (s, 1H, NH), 7.28–7.41 (m, 5H, Ph, Ar, NH), 7.11–7.13 (m, 2H, Ph), 6.56–6.59 (m, 2H, Ar), 5.04 (s, 2H, CH_2_Ph), 3.67–3.71 (m, 2H, CH_2_), 3.53–3.57 (m, 2H, CH_2_), 1.51 (s, 9H, 3 × CH_3_*t*-Bu), 1.49 (s, 9H, 3× CH_3_*t*-Bu); ESI-MS *m/z* found for C_25_H_35_N_5_O_5_: 486.34 [M + H]^+^; RP-HPLC gradient separation from 30% to 100% acetonitrile at 30 min, flow rate: 1 mL/min*, t*_R_ = 13.4 min.

#### 3.6.10. Synthesis of 1-Benzyl-1*H*-*N*-(2-Guanidinoethyl)Pyrrole-3-Carboxamide **15**

From di-Boc guanidine analogue **15d** (50.0 mg, 0.10 mmol) in TFA–DCM 95:5 (3.0 mL), following the general procedure D (1 h) and after chromatographic purification (0.5% NH_4_OH, 19.5% MeOH, 80% DCM), final product **15** (26.5 mg, 91%) was obtained as a white solid. Data for 15: ^1^Η NMR (400 MHz, CD_3_OD) δ 7.27–7.36 (m, 4H, Ph, Ar), 7.20–7.22 (m, 2H, Ph), 6.78 (dd, 1H, *J* = 2.8, 2.4 Hz, Ar), 6.52 (dd, 1H, *J* = 2.8, 2.0 Hz, Ar), 5.13 (s, 2H, CH_2_Ph), 3.46 (t, 2H, *J* = 6.3 Hz, CH_2_), 3.35 (t, 2H, *J* = 6.3 Hz, CH_2_); ^13^C NMR (100 MHz, CD_3_OD) δ 168.6 (C=O), 159.0 (C=NH), 139.0 (C Ph), 129.8 (2× CH Ph), 129.0 (CH Ph), 128.5 (2× CH Ph), 125.4 (CH Ar), 123.6 (CH Ar), 120.1 (C Ar), 109.0 (CH Ar), 54.5 (CH_2_Ph), 42.4 (CH_2_), 39.4 (CH_2_); ESI-MS *m/z* found for C_15_H_19_N_5_O: 286.66 [M + H]^+^, 243.21 [M–(C(NH)NH_2_) + H]^+^, 214.16 [M–(CH_2_NHC(NH)NH_2_) + H]^+^; RP-HPLC gradient separation from 10% to 100% acetonitrile at 30 min, flow rate: 1 mL/min*,* *t*_R_ = 19.3 min, *R_f_*_=_ 0.46 (MeOH–DCM 2:8).

#### 3.6.11. Synthesis of Methyl 1-Benzyl-1*H*-Pyrrole-2-Carboxylate **16a** [67]

From methyl 1*H*-pyrrole-2-carboxylate (151 mg, 1.21 mmol) and NaH 60% (58.1 mg, 2.42 mmol) in DMF (6.0 mL), and a solution of benzyl bromide (0.21 mL, 1.80 mmol) in DMF (9.0 mL), following the general procedure A (3 h) and after chromatographic purification (DCM), **16a** (234 mg, 90%) was obtained as a pale yellow oil. Data for **16a**: ^1^Η NMR (400 MHz, CDCl_3_) δ 7.23–7.34 (m, 3H, Ph), 7.11 (d, 2H, *J* = 7.2 Hz, Ph), 7.02 (dd, 1H, *J* = 3.4, 1.6 Hz, Ar), 6.89 (app t, 1 H, *J* = 1.6 Hz, Ar), 6.19 (app t, 1H, *J* = 3.4 Hz, Ar), 5.57 (s, 2H, CH_2_Ph), 3.77 (s, 3H, OMe); ESI-MS *m/z* found for C_13_H_13_NO_2_: 216 [M + H]^+^, 138 [(M–Ph) + H]^+^; RP-HPLC gradient separation from 30 to 100% acetonitrile at 30 min, flow rate: 1 mL/min*, t*_R_ = 13.9 min.

#### 3.6.12. Synthesis of 1-Benzyl-1*H*-Pyrrol-2-Carboxylic Acid **16b**

From methyl 1-benzyl-1*H*-pyrrole-2-carboxylate **16a** (230 mg, 1.07 mmol) in MeOH–H_2_O (16.0 mL) and an aq solution of KOH 30% (16.0 mL), following the general procedure B (3 h) and after precipitation, the crude of **16b** (172 mg, 80%) was used in the next step without further purification. Data for **16b**: ^1^Η NMR (400 MHz, CDCl_3_) δ 7.24–7.33 (m, 3 H, Ph), 7.14 (dd, 1H, *J* = 3.8, 2.0 Hz, Ar), 7.11 (d, 2H, *J* = 6.8 Hz, Ph), 6.93 (app t, 1H, *J* = 2.0 Hz, Ar), 6.21 (dd, 1H, *J* = 3.8, 2.8 Hz, Ar), 5.56 (s, 2H, CH_2_Ph); ESI-MS (EI) *m/z* found for C_12_H_11_NO_2_: 240 [M + K]^+^, 224 [M + Na]^+^, 202 [M + H]^+^; RP-HPLC gradient separation from 5% to 100% acetonitrile at 30 min, flow rate: 1 mL/min*, t*_R_ = 16.2 min.

#### 3.6.13. Synthesis of 1-Benzyl-1*H*-*N*-[2-(Tert-Butoxycarbonyl)Aminoethyl]Pyrrole-2-Carboxamide **16c**

From 1-benzyl-1*H*-pyrrole-2-carboxylic acid **16b** (172 mg, 0.86 mmol) in DCM (17 mL), DMAP (21.0 mg, 0.17 mmol), *N*-Boc-ethylenediamine (0.13 mL, 0.86 mmol), and then DCC (266 mg, 1.29 mmol), following the general procedure C (16 h) and after chromatographic purification (20% MeOH-CH_2_Cl_2_), **16c** (248 mg, 84%) was obtained as a clear gum. Data for **16c**: ^1^Η NMR (400 MHz, CDCl_3_) δ 7.20–7.30 (m, 5H, Ph, Ar, NH), 7.11 (d, 2H, *J* = 7.2 Hz, Ph), 6.79 (br s, 1H, Ar), 6.64 (br d, 1H, *J* = 2.0 Hz, NH), 6.13 (app t, 1H, *J* = 3.2 Hz, Ar), 5.60 (s, 2H, CH_2_Ph), 3.42 (t, 2H, *J* = 5.6 Hz, CH_2_), 3.29 (app t, 2H, *J* = 5.6 Hz, CH_2_), 1.43 (s, 9H, 3× CH_3_*t*-Bu); ESI MS *m/z* found for C_19_H_25_N_3_O_3_: 367 [M + Na]^+^, 344 [M + H]^+^, 288 [(M–Ph) + Na]^+^, 244 [(M–Boc) + H]^+^; RP-HPLC gradient separation from 30% to 100% acetonitrile at 30 min, flow rate: 1 mL/min, *t*_R_ = 13.3 min.

#### 3.6.14. Synthesis of 1-Benzyl-1*H*-*N*-[2-(2,3-Di-Tert-Butoxycarbonyl)Guanidinoethyl]Pyrrole-2-Carboxamide **16d**

From *N*-Boc analogue **16c** (248 mg, 0.72 mmol) in TFA–DCM 95:5 (14.4 mL), following the general procedure D (1 h), the crude of 2-(1-benzyl-1*H*-pyrrole-2-carboxamido)ethanaminium 2,2,2-trifluoroacetate **16c’** was dissolved in MeOH–DCM (14.4 mL). Then, from *N*,*N*′-di-(*tert*-butoxycarbonyl)thiourea (299 mg, 1.08 mmol), DIPEA (0.50 mL, 2.88 mmol), and *N*-iodosuccinimide (243 mg, 1.08 mmol), following the general procedure E (~24 h) and after chromatographic purification (20% MeOH-CH_2_Cl_2_), **16d** (136 mg, 39%) was obtained as a clear gum. Partial data for **16c’**: ESI-MS *m/z* found for C_14_H_17_N_3_O: 267 [M + Na]^+^, 244 [M]^+^, 227 [M–NH_2_]^+^, 184 [M–HN(CH_2_)NH_2_]^+^, 158 [M–(CO)HN(CH_2_)NH_2_]^+^; RP-HPLC gradient separation from 5% to 100% acetonitrile at 30 min, flow rate: 1 mL/min*, t*_R_ = 11.1 min. Data for **16d**: ^1^Η NMR (400 MHz, CD_3_OD) δ 7.28–7.18 (m, 3H, Ph), 7.07 (d, 2H, *J* = 6.8 Hz, Ph), 6.92 (dd, 1 H, *J* = 2.4, 1.6 Hz, Ar), 6.76 (dd, 1H, *J* = 3.6, 1.6 Hz, Ar), 6.12 (dd, 1H, *J* = 3.6, 2.4 Hz, Ar), 5.57 (s, 2H, CH_2_Ph), 3.29–3.32 (m, 2H, CH_2_), 3.16 (t, 2H, *J* = 6.2 Hz, CH_2_), 1.52 (s, 9 H, 3× CH_3_*t*-Bu), 1.42 (s, 9H, 3× CH_3_*t*-Bu); ESI-MS *m/z* found for C_25_H_35_N_5_O_5_: 486 [M + H]^+^; RP-HPLC gradient separation from 5% to 100% acetonitrile at 30 min, flow rate: 1 mL/min*, t*_R_ = 21.9 min.

#### 3.6.15. Synthesis of 1-Benzyl-1*H*-*N*-(2-Guanidinoethyl)-Pyrrole-2-Carboxamide **16**

From di-Boc guanidine analogue **16d** (114 mg, 0.23 mmol) in TFA–DCM 95:5 (7.0 mL), following the general procedure E (1 h) and after chromatographic purification (0.5% NH_4_OH, 19.5% MeOH, 80% DCM), final product **16** (58.6 mg, 88%) was obtained as a white solid. Data for **16**: ^1^Η NMR (600 MHz, CD_3_OD) δ 7.19–7.27 (m, 3 H, Ph), 7.07 (d, 2H, *J* = 7.8 Hz, Ph), 6.97–6.98 (m, 1H, Ar), 6.79–6.80 (m, 1H, Ar), 6.14–6.15 (m, 1 H, Ar), 5.59 (s, 2H, CH_2_Ph), 3.40 (t, 2 H, *J* = 6.3 Hz, CH_2_), 3.26 (t, 2H, *J* = 6.3 Hz, CH_2_); ^13^C NMR (100 MHz, CD_3_OD) δ 165.2 (C=O), 159.9 (C=NH), 140.6 (C Ph), 129.5 (2× CH Ph), 129.3 (CH Ph), 128.3 (CH Ar), 127.9 (2× CH Ph), 126.0 (C Ar), 114.9 (CH Ar), 109.0 (CH Ar), 52.7 (CH_2_Ph), 42.4 (CH_2_), 39.3 (CH_2_); ESI-MS *m/z* found for C_15_H_19_N_5_O: 286 [M + H]^+^; RP-HPLC gradient separation from 5% to 100% acetonitrile at 30 min, flow rate: 1 mL/mi, *t*_R_ = 20.2 min, *R_f_* = 0.46 (MeOH–DCM 2:8).

#### 3.6.16. Synthesis of *N*-[2-(2,3-Di-Tert-Butoxycarbonyl)Guanidinoethyl]-1-(*m*-(1-Trityl-Tetrazol-5-yl)Benzyl)-1*H*-Pyrrole-3-Carboxamide **17b** [24]

From 1*H*-pyrrole **21** (65.1 mg, 0.26 mmol), NaH 60% (15.4 mg, 0.39 mmol) in DMF (2.6 mL), and a solution of **17a** [69] (124 mg, 0.26 mmol) in DMF (2.6 mL), following the general procedure A (2 h) and after chromatographic purification (10–100% AcOEt-Et_2_O), **17b** (81.8 mg, 40%) was obtained as a white solid. Data for **17b**: ^1^Η NMR (600 MHz, CDCl_3_) δ 11.48 (s, 1H, NH), 8.06 (d, 1H, *J* = 7.8 Hz, Ar’), 7.99 (s, 1H, Ar’), 7.32–7.42 (m, 13H, Ar, Ar’, Trt, NH), 7.13–7.16 (m, 8H, Ar, Ar’, Trt), 6.60 (d, 1H, *J* = 1.8 Hz, Ar), 5.09 (s, 2H, CH_2_Ar), 3.76 (br s, 2H, CH_2_), 3.59 (br s, 2H, CH_2_), 1.51 (s, 9H, 3× CH_3_ *t*-Bu), 1.49 (s, 9H, 3× CH_3_ *t*-Bu); ESI-MS *m/z* found for C_45_H_49_N_9_O_5_: 796.30 [M + H]^+^, 696.27 [(M–Boc) + H]^+^, 341.71 [(17a–Trt) + Boc + H]^+^, 243 [Trt]^+^; RP-HPLC gradient separation from 60% to 100% acetonitrile at 30 min, flow rate: 1 mL/min, *t*_R_ = 15.8 min.

#### 3.6.17. Synthesis of 1-(2-(1-(*m*-(1*H*-Tetrazol-5-Yl)Benzyl)-1*H*-Pyrrole-3-Carboxamido)Ethyl) Guanidinium 2,2,2-Trifluoroacetate **17**

From **17b** (20 mg, 0.025 mmol) and TES (0.004 mL, 0.025 mmol) in TFA–DCM 95:5 (0.75 mL), following the general procedure D (5 h) and after purification by semi-preparative HPLC (10–60% ACN, 45 min), then lyophilization, final product **17** (7.89 mg, 68%) was obtained as a white solidwith 99% purity. Data for **17**: ^1^Η NMR (600 MHz, CD_3_OD) δ 7.95 (d, 1H, *J* = 7.8 Hz, Ar’), 7.90 (s, 1H, Ar’), 7.57 (t, 1H, *J* = 7.8 Hz, Ar’), 7.43 (d, 1H, *J* = 7.8 Hz, Ar’), 7.41 (app dd, 1H, *J* = 2.4, 1.8 Hz, Ar), 6.85 (dd, 1 H, *J* = 3.0, 2.4 Hz, Ar), 6.56 (dd, 1H, *J* = 3.0, 1.8 Hz, Ar), 5.26 (s, 2H, CH_2_Ar’), 3.47 (t, 2H, *J* = 6.0 Hz, CH_2_), 3.36 (t, 2H, *J* = 6.0 Hz, CH_2_); ^13^C NMR (100 MHz, CD_3_OD) δ 168.6 (C=O), 159.0 (2× C=NH), 140.8 (C Ar’), 131.5 (CH), 131.1 (CH), 127.7 (CH), 127.2 (CH), 126.3 (C Ar’), 125.4 (CH), 123.7 (CH), 120.5 (C Ar), 109.4 (CH Ar), 54.0 (CH_2_Ar’), 42.4 (CH_2_), 39.5 (CH_2_); ESI-MS *m/z* found for C_16_H_19_N_9_O: 354.53 [Μ + Η]^+^; RP-HPLC gradient separation from 10% to 100% acetonitrile at 30 min, flow rate: 1 mL/min, *t*_R_ = 17.7 min.

#### 3.6.18. Synthesis of *N*-[2-(2,3-Di-Tert-Butoxycarbonyl)Guanidinoethyl]-1-(*p*-Tert-Butoxycarbonyl Methyl)Benzyl-1*H*-Pyrrole-3-Carboxamide **18b**

From 1*H*-pyrrole **21** (168 mg, 0.66 mmol), NaH 60% (39.8 mg, 1.66 mmol) in DMF (6.6 mL), and a solution of **18a**, (189 mg, 0.66 mmol) in DMF (6.6 mL), following the general procedure A (2 h) and after chromatographic purification (50–100% AcOEt-Et_2_O), **18b** (87.6 mg, 22%) was obtained as a white solid). Data for **18b**: ^1^Η NMR (400 MHz, CDCl_3_) δ 11.49 (s, 1H, NH), 8.78 (s, 1H, NH), 7.46 (s, 1H, NH), 7.31 (s, 1H, Ar), 7.22 (d, 2H, *J* = 8.0 Hz, Ar’), 7.07 (d, 2H, *J* = 8.0 Hz, Ar’), 6.57 (d, 2H, *J* = 2.0 Hz, Ar), 5.02 (s, 2H, CH_2_Ar), 3.67–3.71 (m, 2 H, CH_2_), 3.56 (br s, 2H, CH_2_), 3.50 (s, 2H, CH_2_CO_2_*t*-Bu), 1.51 (s, 9H, 3× CH_3_*t*-Bu),1.49 (s, 9H, 3× CH_3_*t*-Bu), 1.43 (s, 9H, 3× CH_3_*t*-Bu).

#### 3.6.19. Synthesis of 1-(2-(1-(*p*-(Carboxymethyl)Benzyl)-1*H*-Pyrrole-3-Carboxamido)Ethyl) Guanidinium 2,2,2-Trifluoroacetate **18**

From **18b** (50 mg, 0.083 mmol) and TES (0.01 mL, 0.083 mmol) in TFA–DCM 95:5 (2.50 mL), following the general procedure D (5 h) and after purification by semi-preparative HPLC (10–60% ACN, 45 min), then lyophilization, final product **18** (23.2 mg, 61%) was obtained as a white solidwith 98% purity. Data for **18**: ^1^Η NMR (600 MHz, CD_3_OD) δ 7.33 (app dd, 1H, *J* = 2.4, 1.8 Hz, Ar), 7.27 (d, 2H, *J* = 8.1 Hz, Ar’), 7.17 (d, 2H, *J* = 8.1 Hz, Ar’), 6.78 (dd, 1H, *J* = 3.0, 2.4 Hz, Ar), 6.52 (dd, 1H, *J* = 3.0, 1.8 Hz, Ar), 5.11 (s, 2H, CH_2_Ar’), 3.59 (s, 2H, CH_2_CO_2_H), 3.46 (t, 2H, *J* = 6.0 Hz, CH_2_), 3.35 (t, 2H, *J* = 6.0 Hz, CH_2_); ESI-MS *m/z* found for C_17_H_21_N_5_O_3_: 344.66 [Μ + Η]^+^; RP-HPLC gradient separation from 10% to 100% acetonitrile at 30 min, flow rate: 1 mL/min, *t*_R_ = 16.6 min.

#### 3.6.20. Synthesis of *N*-[2-(2,3-Di-Tert-Butoxycarbonyl)Guanidinoethyl]-1-(*P*-Methoxycarbonyl) Benzyl-1*H*-Pyrrole-3-Carboxamide **19b**

From 1*H*-pyrrole **21** (109 mg, 0.43 mmol), NaH 60% (25.8 mg, 0.65 mmol) in DMF (4.3 mL), and a solution of **19a**, (98.5 mg, 0.43 mmol) in DMF (4.3 mL), following the general procedure A (2 h) and after chromatographic purification (50–100% AcOEt-Et_2_O), **19b** (103 mg, 44%) was obtained as a white solid. Data for **19b**: ^1^Η NMR (400 MHz, CDCl_3_) δ 11.49 (s, 1H, NH), 8.73 (s, 1H, NH), 7.98 (d, 2H, *J* = 8.2 Hz, Ar’), 7.49 (s, 1H, NH), 7.31 (t, 1H, *J* = 1.8 Hz, Ar), 7.15 (d, 2H, *J* = 8.2 Hz, Ar’), 6.56–6.60 (m, 2 H, Ar), 5.10 (s, 2H, CH_2_Ar), 3.90 (s, 3H, OCH_3_), 3.67–3.71 (m, 2H, CH_2_), 3.55–3.57 (m, 2 H, CH_2_), 1.50 (s, 9 H, 3× CH_3_*t*-Bu), 1.49 (s, 9H, 3× CH_3_*t*-Bu); ESI-MS *m/z* found for C_27_H_37_N_5_O_7_: 344.60 [(Μ–2×Boc) + Η]^+^; RP-HPLC gradient separation from 5% to 100% acetonitrile at 30 min, flow rate: 1 mL/min, *t*_R_ = 25.7 min.

#### 3.6.21. Synthesis of 1-(2-(1-(*p*-(Methoxycarbonyl)Benzyl)-1*H*-Pyrrole-3-Carboxamido)Ethyl) Guanidinium 2,2,2-Trifluoroacetate **19**

From **19b** (20 mg, 0.037 mmol) in TFA–DCM 95:5 (0.74 mL), following the general procedure D (5 h) and after purification by semi-preparative HPLC (10–60% ACN, 45 min), then lyophilization, final product **19** (13.3 mg, 79%) was obtained as a white solid with 99% purity. Data for **19**: ^1^Η NMR (400 MHz, CD_3_OD) δ 7.98 (d, 2H, *J* = 8.0 Hz, Ar’), 7.36 (app t, 1H, *J* = 2.0 Hz, Ar), 7.28 (d, 2H, *J* = 8.0 Hz, Ar’), 6.81–6.82 (m, 1H, Ar), 6.55 (dd, 1H, *J* = 2.8, 2.0 Hz, Ar), 5.23 (s, 2H, CH_2_Ar’), 3.89 (s, 3H, OCH_3_), 3.47 (t, 2H, *J* = 6.4 Hz, CH_2_), 3.35 (t, 2H, *J* = 6.4 Hz, CH_2_); ESI-MS *m/z* found for C_17_H_21_N_5_O_3_: 344.68 [Μ + Η]^+^; RP-HPLC gradient separation from 5% to 100% acetonitrile at 30 min, flow rate: 1 mL/min, *t*_R_ = 18.9 min.

#### 3.6.22. Synthesis of *N*-(2,3-Di-(Tert-Butyloxycarbonyl)Guanidinoethyl)Pyrrole-3-Carboxamide **21**

To a solution of 1*H*-pyrrole-3-carboxylic acid (85.5 mg, 0.77 mmol, 1.00 equiv) in DCM (5 mL) and DMF (1 mL), at 0 °C, HOBt (178 mg, 1.16 mmol, 1.50 equiv) and DCC (239 mg, 1.16 mmol, 1.50 equiv) were added. The mixture was stirred at the same temperature for 10 min, and was then supplemented with a solution of **20** [39] (350 mg, 1.16 mmol, 1.50 equiv) in DCM (13 mL) followed by DIPEA (0.20 mL, 1.16 mmol, 1.50 equiv). The reaction mixture warmed to rt over 3 h and monitored by TLC (10% MeOH in DCM). The solvents were removed in vacuo, and the remaining residue was purified by column chromatography (5% EtOH in Et_2_O) to yield **21** (170 mg, 0.67 mmol, 87%) as a beige solid. Data for **21**:^1^Η NMR (400 MHz, CDCl_3_) δ 11.51 (s, 1H, NH), 8.82 (s, 2H, NH), 7.57 (s, 1H, NH), 7.43–7.44 (m, 1H, Ar), 6.73–6.75 (m, 1H, Ar), 6.63–6.65 (m, 1H, Ar), 3.71–3.75 (m, 2H, CH_2_), 3.57–3.60 (m, 2H, CH_2_), 1.55 (s, 9 H, 3× CH_3_*t*-Bu), 1.51 (s, 9 H, 3× CH_3_*t*-Bu); ESI-MS *m/z* found for C_18_H_29_N_5_O_5_: 341 [M−tBu + H]^+^, 381 [M–tBu + K + H]^2+^; RP-HPLC gradient separation from 5% to 100% acetonitrile at 30 min, flow rate: 1 mL/min, *t*_R_ = 21.4 min.

### 3.7. Molecular Orbital Calculations

Two different approaches were applied in order to calculate the binding energy of the compounds inside TCR, namely density functional theory (DFT) [70] and semi-empirical (SE) methods [71]. For the application of DFT, several variants [72] differing in choice of functional [73] and basis set were implemented in order to calculate the interaction. This procedure was followed to select the most appropriate method for our complex (see Supporting Information). The self-consistent reaction field (SCRF) was used with DFT energies, optimizations, and frequency calculations to model the system in solution (H_2_O). All DFT calculations were performed with Gaussian09 [74]. A similar protocol was applied for the calculation of the interaction energy including the whole TCR with SE methodologies. The MOPAC2012 [71] software was used for the SE calculations. Due to the large size of the protein–ligand systems, the keyword MOZYME [75] was employed to accelerate the calculations, and the COSMO [76] function was used to estimate the effect of the solvent. For the methods including dispersion (D), the optimized parameters for H, N, C, and O, as reported by McNamara and Hillier [72,73], were used. Semi-empirical calculations were performed on the whole complex (ligand–TCR), while DFT on the ligand and selected TCR residues.

### 3.8. In Vitro Evaluation of the Analogues Using Human PBMC

Peripheral blood samples (10 mL) were drawn from two healthy volunteers (one 24-year-old male and one 35-year-old female) and were analyzed in a CELL-DYN Sapphire hematology analyzer (Abbot Diagnostics, Lake Forest, IL, USA) to determine the absolute numbers and percentages of leukocytes, in particular lymphocytes and monocytes. Peripheral blood mononuclear cells (PBMCs) were isolated by centrifugation over a Ficoll–Paque gradient (Biochrom AG, Berlin, Germany) and washed ×3 with ice-cold RPMI1640 culture medium (Gibco BRL, Waltham, MA, USA). The cells were stained with CellTrace CFSE for flow cytometry (Invitrogen-Thermo Fisher Scientific Inc., Waltham, MA, USA) as described and cultured in RPMI1640 (with 10% Fetal Bovine Serum, 50 IU/mL penicillin, 100 μg/mL streptomycin, and 5 × 10^−5^ mol/L mercaptoethanol) (Invitrogen-Thermo Fisher Scientific Inc., Waltham, MA, USA) at a concentration of 10^6^ cells/mL. PBMCs were cultured for three days in the presence of an anti–CD28 antibody (5 μg/mL) (BD Biosciences/Pharmingen, San Diego, CA, USA) and different concentrations of peptide MBP_83–96_ (0.01 nM, 0.1 nM, 1 nM, 10 nM, and 100 nM) to estimate the optimal concentration that induces T cell proliferation. When the optimal MBP_83–96_ concentration was determined, the cultures were repeated as previously with the addition of the same concentration of each of the studied analogues per point, in triplicate. T cell proliferation was monitored and quantified by flow cytometry. Flow cytometric acquisition and analysis were performed on at least 10,000 acquired events per sample using the BD FACSCalibur™ platform.

### 3.9. In Vitro Evaluation of the Analogues Using Mouse-Specific MBP_83–99_ T Cells

Mice, SJL/J females, aged 4–9 weeks were purchased from the Animal Resource Centre (Perth Australia). All mice had free access to food and water, and were housed in a temperature-controlled environment with 12-h day/night cycles at the animal holding room Werribee Campus Animal Facility (Melbourne, Australia). They were allowed to acclimatize for at least 7 days before immunizations. All experiments were completed according to the guidelines of the Australian Code of Practice for the Care and Use of Animals for Scientific Purposes and were approved by Victoria University Animal Experimentation Ethics Committee (AEC15/013). Mice were subcutaneously injected with 50 μg/100 μL reduced mannan conjugated to MBP_83–99_ via a 10 amino acid linker (KG)_5_ as previously described [34,77]. This conjugate has been shown to induce T cell proliferation to native peptide MBP_83–99_ [26,27,30,31,32,34,77]. Spleen cells from 3 immunized SJL/J mice were isolated 10 days after immunization and assessed by T cell proliferation assay. As we have previously shown that the native peptide MBP_83–99_ conjugated to mannan induces strong proliferative T cells to recall MBP_83–99_ peptide, we used 3 mice/group to test each of the compounds’ ability to inhibit this T cell proliferation. Hence, 3 mice/group in this screening process are adequate for determining the optimal compound for inhibiting T cell proliferation. Spleen cells at 2 × 10^5^ in 100 µL of culture media were seeded into 96 well U-bottom plates and incubated for 1–6 days at 37 °C in the presence of recall MBP_83__–__99_ peptide (10 nM) with or without 100x molar excess of compounds **15**–**19** or **AMB**. Proliferation was assessed by the addition of MTT (3-(4,5-dimethylthiazol-2-yl)-2,5-diphenyltetrazolium bromide, a tetrazole) for 6 hours and proliferation assessed via spectrophotometry (Biorad microplate reader, 6.0) using a wavelength of 570 nm. All experiments were conducted in triplicate. The percentage of inhibition of cell proliferation in the presence of compound was calculated and plotted.

## 4. Conclusions

A ligand-based pharmacophore model was developed based on the conformational properties of the dominant MBP_83–96_ epitope in complex with the TCR. The resulting model was employed for the virtual screening of the ZINC database for potential hits. A subset of the database, containing 500,000 all clean/ commercially available compounds, were screened, and the search yielded 13 hits. The potential inhibitors were ranked according to their inhibitory activity against TCR with the employment of molecular docking simulations. The compound with the highest docking score (compound **10**) was selected as lead and was subjected to optimization via chemical modifications. The resulting optimized molecule (compound **14**) presented increased docking score to the TCR and improved chemical properties such as TPSA and logP (Table 2).

The conformational analysis and the positioning of compound **14** in the TCR binding pocket led to the further modification with the addition of a methylene group and the organic synthesis of two isomers (compounds **15** and **16**). The analysis of the conformational properties of the three analogues via MD simulation experiments showed that analogue **15** has the most optimal positioning inside the TCR binding cavity and is better tethered within the receptor (Figure 5a). Extensive MD simulations may offer a deeper understanding of the interactions between the designed analogues and the receptor, and prove to be a valuable tool in drug design. Furthermore, the interaction energy between the potential inhibitor (compound **15**) and the TCR was explored by employing a variety of molecular orbital approaches. DFT and SE methodologies were used in order to calculate the interaction energy between selected residues of the TCR, as well as the entire TCR, and the proposed inhibitor **15**. The combination of the two methodologies allows us to identify whether only certain residues have the greatest impact in the binding of compound **15** or other conformational aspects of the TCR are important in its binding. The agreement between the DFT and the SE methods show that the binding of the potential inhibitor to the TCR is attributed only to the residues surrounding the binding cavity and not to other conformational changes observed in the TCR. The results of the in vitro evaluation (Figure 8) suggest that both analogues **15** and **16** may serve as good candidate antagonists to be developed further for the inhibition of proliferation of T-cells that recognize the MBP_83–96_ antigen.

## Figures and Tables

**Table 1:** Chemical structure and docking scores of the proposed potential T cell receptor (TCR) antagonists (compounds **1**–**19**).

Compound Number	Structure	*ΔG* ^d^ (kcal/mol)
MBP_83–96_	Seq: ENPVVHFFKNIVTP	−11.89
**1** ^a^	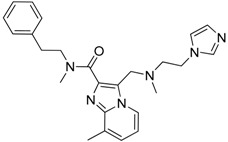	−15.87
**2** (* S/** R)	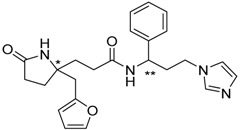	−19.71
**3** (* S/** S)	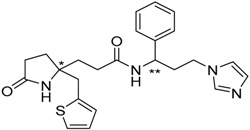	−14.46
**4** (* R)	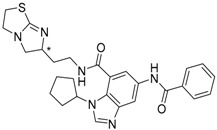	−14.43
**5**	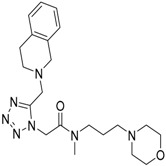	−10.32
**6**	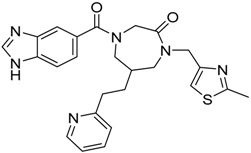	−15.34
**7**	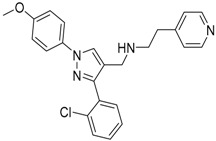	−16.38
**8** (* S)	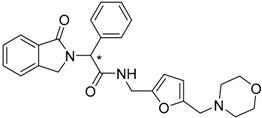	−13.26
**9**	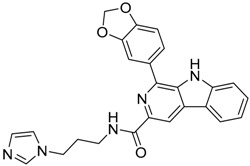	−15.86
**10** (*Lead Compound*)	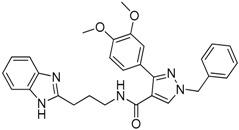	−21.56
**11** (* R)	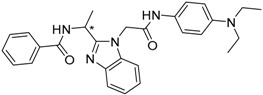	−20.85
**12**	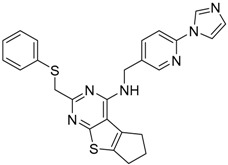	−16.05
**13** (* S)	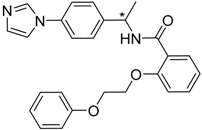	−20.65
**14** ^b^	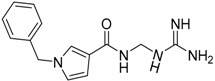	−23.76
**15**	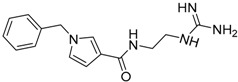	−18.13
**16**	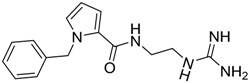	−18.03
**17** ^c^	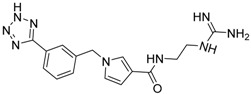	−18.49
**18**	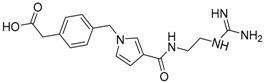	−20.70
**19**	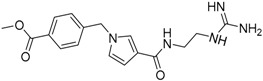	−21.32

^a^ Compounds **1**–**13** were obtained from the pharmacophore model. ^b^ Compounds **14**–**16** were derived through modifications of the lead compound **10**. ^c^ Compounds **17**–**19** were modified analogues of compound **15**. ^d^ Docking score as calculated by the MOE2016 software at 298 K.

**Table 2:** Properties of lead compound **10** and optimized analogues **14**–**19**.

Compound	MW (g/mol)	TPSA (Å^2^)	logP	Docking Score (kcal/mol)
**10**	495.57	94.06 ^a^	5.25 ^b^	−21.56
**14**	272.33	97.67	−0.84	−23.76
**15**	286.36	97.67	−0.84	−18.13
**16**	286.36	97.67	−0.71	−18.03
**17** ^c^	354.40	152.13	−1.62	−18.49
**18**	344.39	137.34	−1.49	−20.70
**19**	344.39	126.34	−1.42	−21.32

^a,b^ Total Polar Surface Area (TPSA) and hydrophobicity content (logP) values are reported as shown on Ambinter Chemicals catalogue: http://www.ambinter.com/. ^c^ Compounds **17**–**19** are modified analogues of compound **15**.

**Table 3:** Hydrogen bonds for all optimized analogues (**14**–**19**) as reported in the docking and MD simulation experiments.

TCR Residues	Compounds											
	14		15		16		17		18		19	
	Dock	MD	Dock	MD	Dock	MD	Dock	MD	Dock	MD	Dock	MD
AsnA30	✓ ^a^		✓	✓			✓			✓		✓
AspA92	✓		✓	✓	✓		✓	✓	✓	✓	✓	✓
ThrA93												✓
GlyA96	✓		✓	✓	✓	✓	✓	✓	✓	✓		✓
ThrA97	✓			✓			✓	✓				
TyrA98							✓			✓		
TyrA100		✓										
AsnB51									✓		✓	
LysB55									✓		✓	
SerB101						✓		✓				
AlaB103						✓						
AsnB104		✓										
GluB106		✓										

^a^ presence of hydrogen bonds.

## Supplementary Materials

Supplementary materials can be found at www.mdpi.com/1422-0067/18/6/1215/s1.

## Abbreviations

CDRs	complementarity determining regions
DCC	*N*,*N′*-dicyclohexylcarbodiimide
DCM	dichloromethane
DIPEA	*N*,*N*-diisopropylethylamine
DCU	dicyclohexylurea
DMAP	4-dimethylaminopyridine
DMF	dimethylformamide
ESI MS	electrospray ionization mass spectrometry
HLA	human leukocyte antigen
HOBt	1-hydroxybenzotriazole
MBP	myelin basic protein
MD	molecular dynamics
MHC	major histocompatibility complex
MS	multiple sclerosis
MW	molecular weight
^1^Η NMR	proton nuclear magnetic resonance
^13^C NMR	carbon-13 nuclear magnetic resonance
PBMC	peripheral blood mononuclear cells
RP-HPLC	reversed phase high-performance liquid chromatography
TCR	T cell receptor
TES	triethylsilane
TFA	trifluoroacetic acid
Th	T helper
TLC	thin layer chromatography
TPSA	total polar surface area
