# Supplementary material for: Design and Synthesis of Non-Peptide Mimetics Mapping the Immunodominant Myelin Basic Protein (MBP83–96) Epitope to Function as T-Cell Receptor Antagonists
Source: Int J Mol Sci. 2017 Jun 8;18(6):1215. doi: 10.3390/ijms18061215 (PMC5486038; doi:10.3390/ijms18061215)
Supplement: Supplementary file 1 [file ijms-18-01215-s001.pdf]

Supplementary Information

Design and Synthesis of Non-Peptide Mimetics Mapping the Immunodominant Myelin Basic Protein (MBP_{83-96}) Epitope to Function as T-cell Receptor Antagonists

Mary-Patricia Yannakakis [1,2], Carmen Simal [1], Haralambos Tzoupis [1], Maria Rodi [3], Narges Dargahi [4], Monica Prakash [4], Athanasia Mouzaki [3], James A. Platts [2], Vasso Apostolopoulos [4,*,†] and Theodore V. Tselios [1,*,†]

[1] Department of Chemistry, University of Patras, Rion Patras 26504, Greece; yannakakism@gmail.com (M.-P.Y.); carmen.simal@gmail.com (C.S.); haralambostz@gmail.com (H.T.)
[2] School of Chemistry, Cardiff University, Park Place, Cardiff CF103AT, Wales, UK; platts@cardiff.ac.uk
[3] Laboratory of Immunohematology, Division of Hematology, Department of Internal Medicine, Medical School, University of Patras, Rion, Patras 26500, Greece; marodi_biol@yahoo.gr (M.R.); mouzaki@upatras.gr (A.M.)
[4] Centre for Chronic Disease, College of Health and Biomedicine, Victoria University, VIC 3021, Australia; Narges.dargahi@live.vu.edu.au (N.D.); monica.prakash@vu.edu.au (M.P.)
* Correspondence: vasso.apostolopoulos@vu.edu.au (V.A.); ttselios@upatras.gr (T.V.T.); Tel.: +61-3-9919-2025 (V.A.); +30-261-099-7905 (T.V.T.)
† These authors have equal contribution.

Academic Editor: Christoph Kleinschnitz
Received: 26 April 2017; Accepted: 2 June 2017; Published: date

(a)

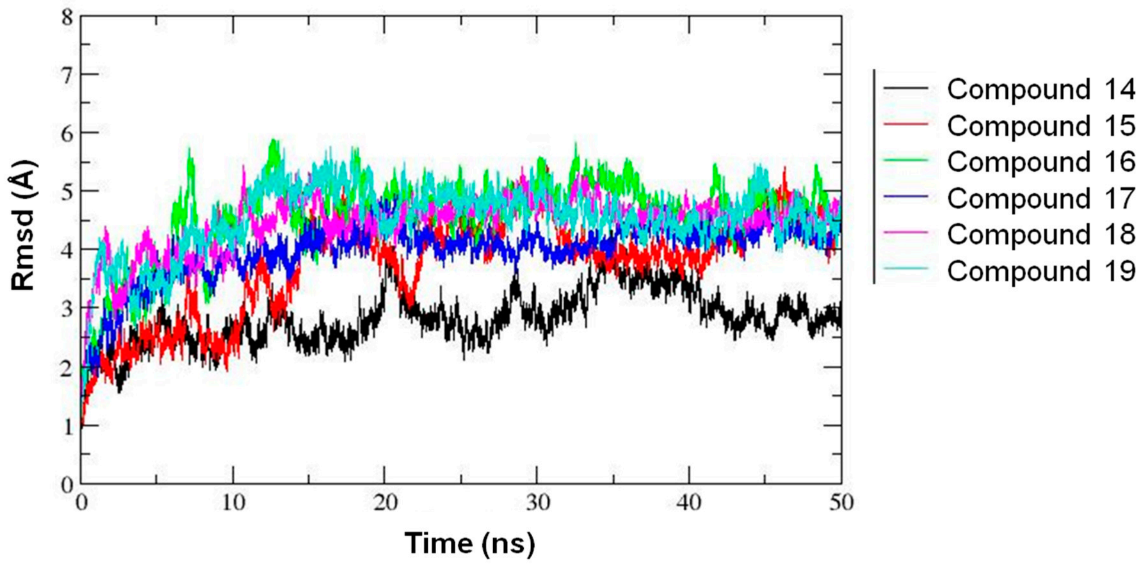
Rmsd (Å)
Time (ns)
0
1
2
3
4
5
6
7
8
0
10
20
30
40
50
Compound 14
Compound 15
Compound 16
Compound 17
Compound 18
Compound 19

(b)

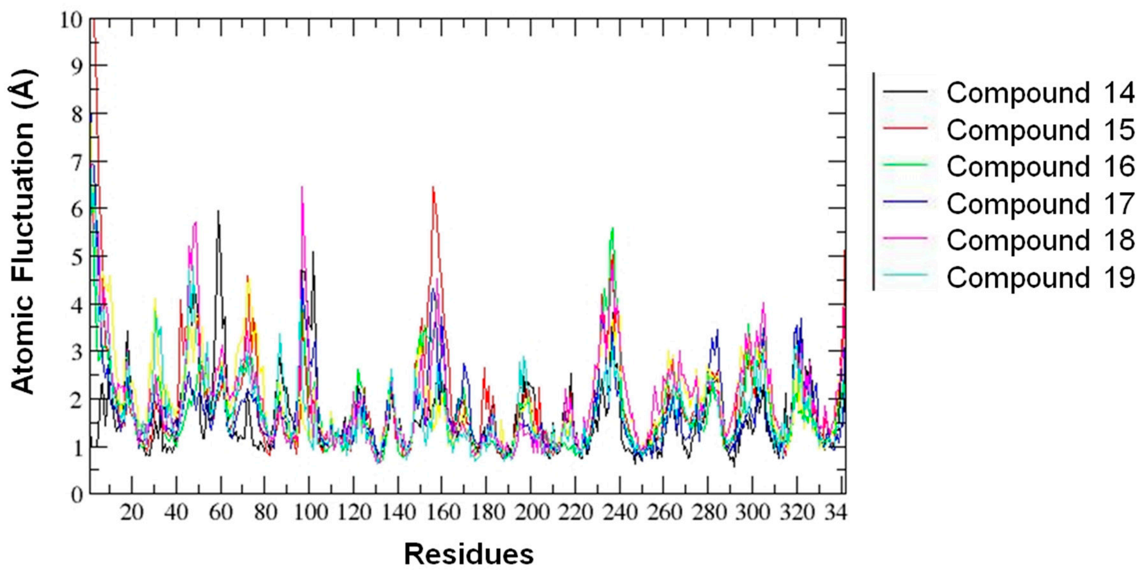
Atomic Fluctuation (Å)
Residues
0
1
2
3
4
5
6
7
8
9
10
20
40
60
80
100
120
140
160
180
200
220
240
260
280
300
320
34
Compound 14
Compound 15
Compound 16
Compound 17
Compound 18
Compound 19

(c)

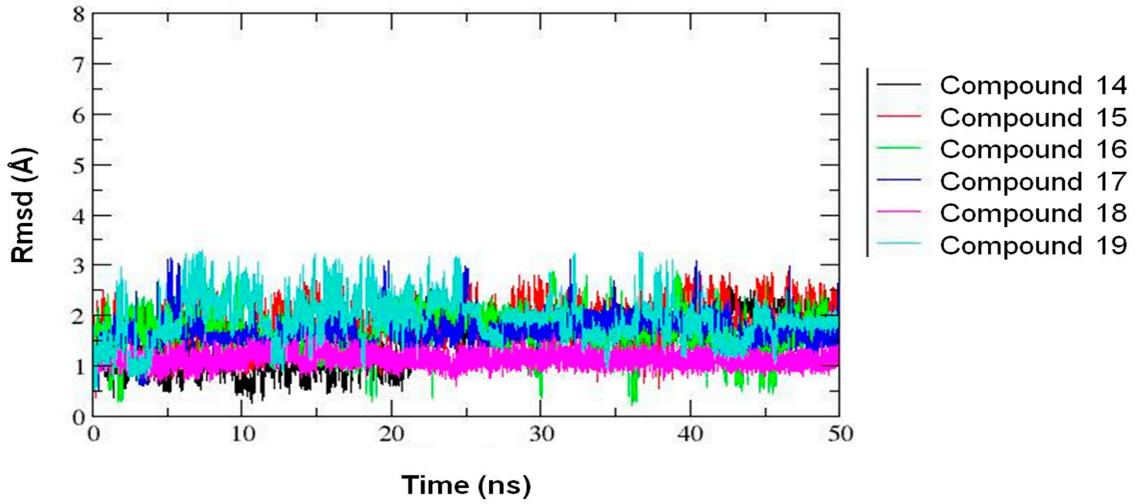

Figure S1. (**a**) Plot of rms values over time for the Cα atoms of T cell receptor (TCR) residues in complex with compounds **14-19**, (**b**) atomic positional fluctuations for the different residues in the TCR for the different molecular dynamics (MD) simulation runs, and (**c**) rms values for compounds **14-19**, during the MD simulations.

Table S1. The chemical structures and properties of compounds **1-13**, purchased from Ambinter Chemicals.

Ambinter Code	Structure	Molecular Weight	logP[a]	PSA[b]
**Amb11063559** $C_{25}H_{30}N_6O$	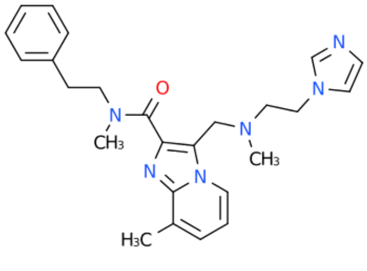	430.545	3.2859	58.67
**Amb10213450** $C_{24}H_{28}N_4O_3$	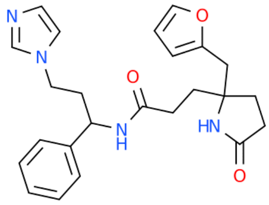	420.504	4.1151	89.16

**Amb11124336** $C_{24}H_{28}N_4O_2S$	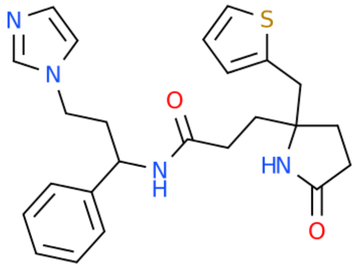	436.57	4.5836	104.26
**Amb11124920** $C_{27}H_{26}N_6O_2S$	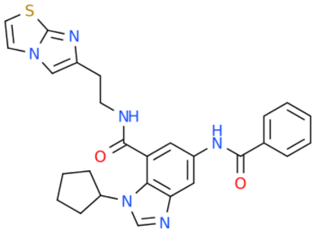	498.599	5.5493	121.56
**Amb11049469** $C_{21}H_{31}N_7O_2$	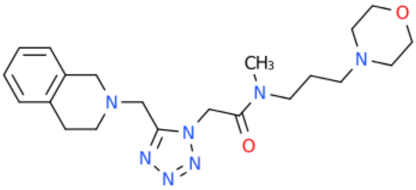	413.517	0.2879	79.62
**Amb11020966** $C_{24}H_{24}N_6O_3S$	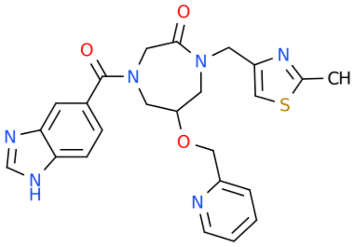	476.551	2.6686	132.55
**Amb11084608** $C_{24}H_{23}ClN_4O$	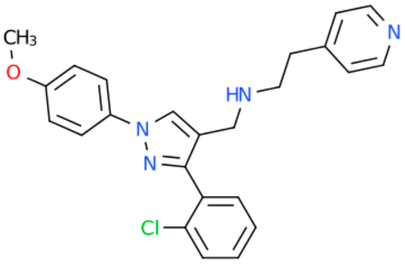	418.919	5.3195	51.97
**Amb20310491** $C_{26}H_{27}N_3O_4$	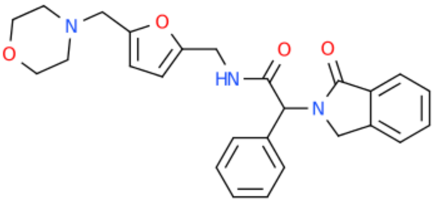	445.51	3.392	75.02

**Amb562959** $C_{25}H_{21}N_5O_3$	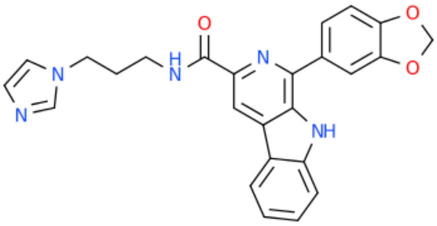	439.466	4.5193	94.06
**Amb499010** $C_{29}H_{29}N_5O_3$ **(LEAD MOLECULE)**	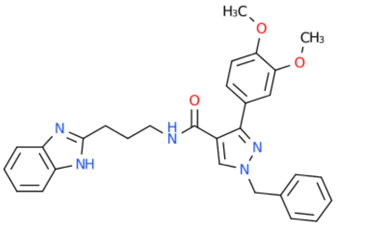	495.572	5.2454	94.06
**Amb409596** $C_{28}H_{31}N_5O_2$	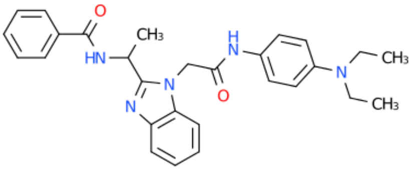	469.578	5.4761	79.26
**Amb509000** $C_{25}H_{22}N_6S_2$	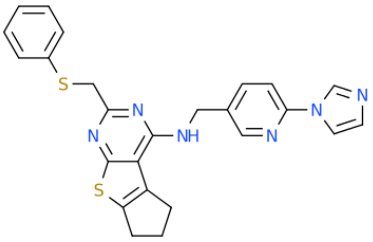	470.612	5.7381	122.06
**Amb58395** $C_{26}H_{25}N_3O_3$	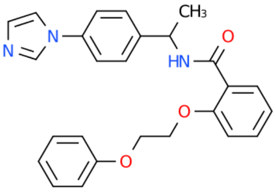	427.495	5.212	65.38

[a,b] logP and PSA values are reported as shown on Ambinter Chemicals catalogue: (http://www.ambinter.com/)

Table S2. Semi-empirical (SE) binding energy in solvent, for molecule **15** in complex with the whole TCR and with selected binding site residues (kcal/mol).

Method	Whole Receptor	Selected Site Residues
**PM7**	-34.39	-24.09
**PM6-D**	6.00	0.26
**PM6-DH2**	-0.57	-6.16
**AM1**	19.30	16.41
**PM6**	27.89	18.178
**RM1**	28.60	23.21
**PM3**	25.65	18.71
**PM6-D3**	-1.02	-6.21
**PM6-DH+**	-1.56	-5.87

Table S3. Interaction energies for 20 MD snapshots of analogue **15**, using PM7 in solvent.

MD Snapshot	Analogue 15 Interaction Energy (Kcal/mol)
1	-53.6571
2	-68.5798
3	-51.6185
4	-52.5194
5	-43.5009
6	-47.5047
7	-46.076
8	-43.8191
9	-39.9552
10	-55.3105
11	-48.8477
12	-50.9680
13	-28.8528
14	-44.3055
15	-45.3845
16	-42.7999
17	-52.5803
18	-38.2581
19	-44.1766
20	-48.007
**Mean**	-47.2926
**Standard Deviation**	7.9095
**Standard Error (SE)**	1.77

Table S4. Interaction energy for molecule **15** in solvent as calculated by different density functional theory (DFT) methodologies, employing different basis sets.

DFT Method in Solvent	Basis Set					
	**cc-pVTZ**	**cc-pVDZ**	**6-311G**	**6-31G**	**6-31G****	**6-31+G****
**B3lyp**	-10.88	-24.40	-18.46	-23.84	-23.78	-6.66
**Cam-b3lyp**	-18.58	-32.12	-26.74	-31.53		
**M06**	-33.25	-40.09		-38.58		
**M06-2X**	-31.63	-40.75				
**B97D**	-42.41			-54.58		
**MPW1PW91**	-11.33			-22.23		
**BHandH**	-41.77			-51.86		
**B3lyp-D**	-42.85					

Table S5. Interaction energies for analogues **17-19**, using PM7.

**Compounds**	**Interaction Energy (Kcal/mol)**
**17**	-35.39
**18**	-37.28
**19**	-35.40

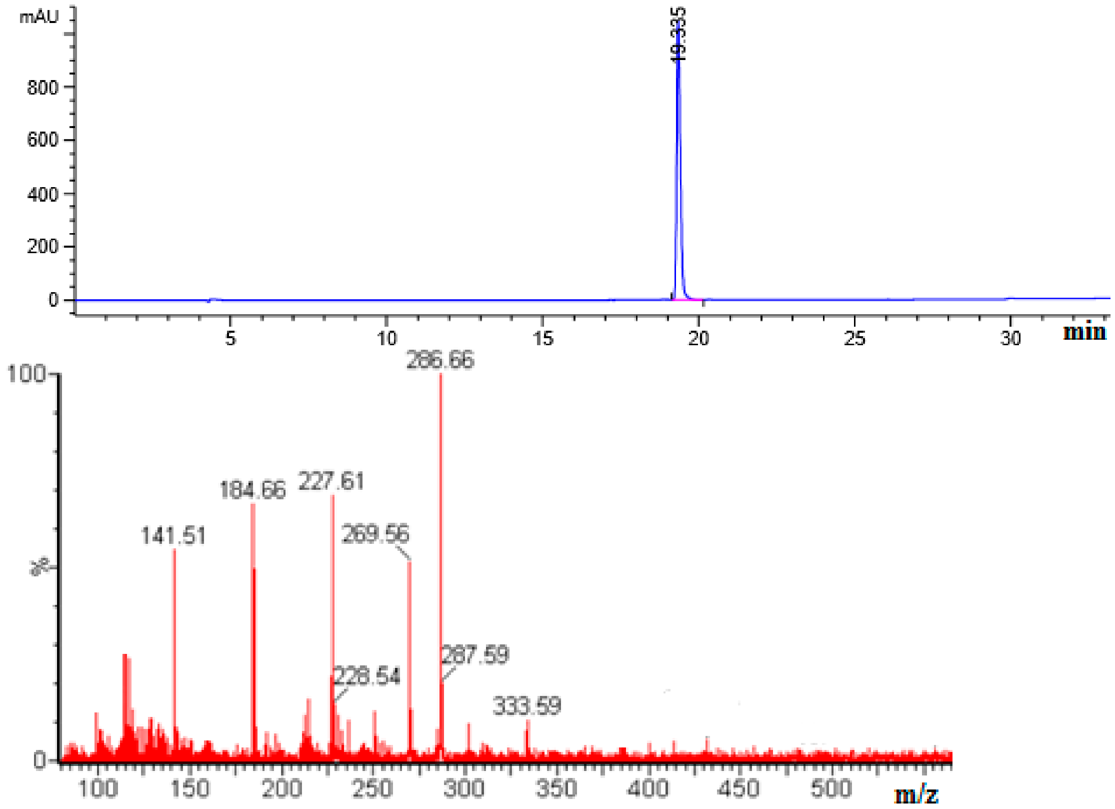

Figure S2. RP-HPLC chromatogram (top) and ESI-MS (bottom) of final analogue **15** ($MW_{theoretical}$: 285.34).
RP-HPLC Conditions:
i) Column: Agilent ZORBAX Eclipse Plus C18 (3.5μm, 100x4.6mm),
ii) Solvents: H_2O (0.08% TFA), AcN (0.08%TFA),
iii) Gradient elution: from 10% AcN to 100% AcN over 30min.
iv) t_R: 19.3 min, Purity: 99%

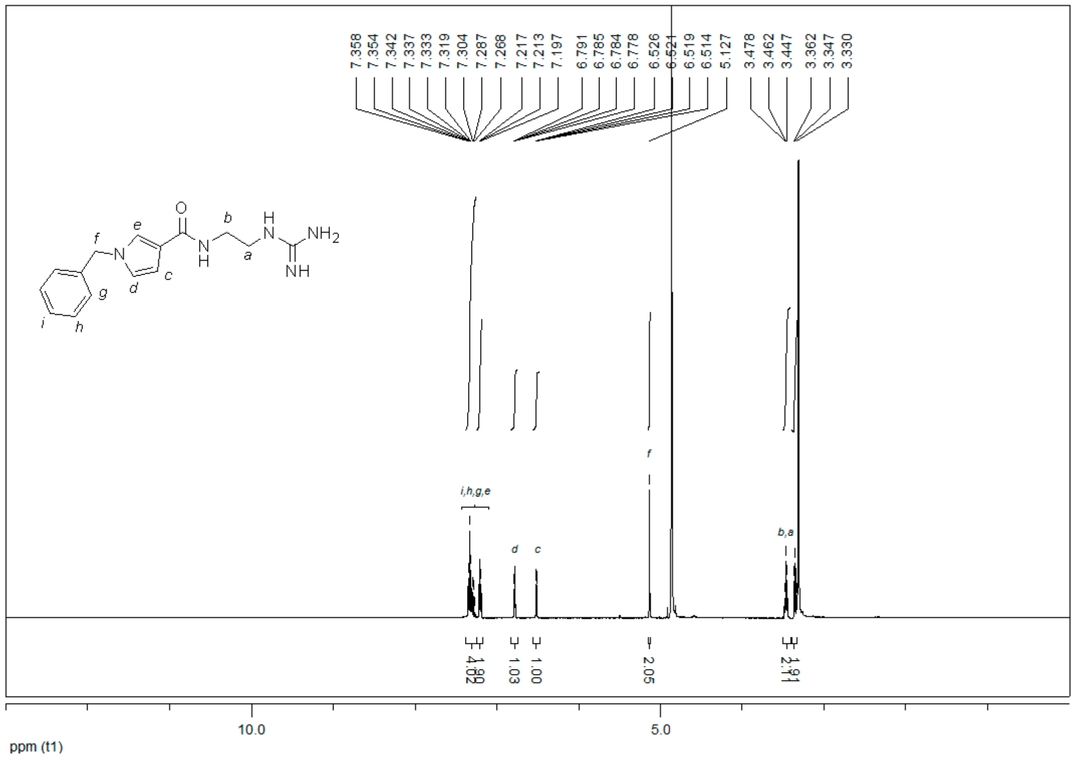

Figure S3. ¹H NMR spectra of analogue **15** (400 MHz, CD_3OD) δ 7.27-7.36 (m, 4 H, Ph, Ar), 7.20-7.22 (m, 2 H, Ph), 6.78 (dd, 1 H, *J* = 2.8, 2.4 Hz, Ar), 6.52 (dd, 1 H, *J* = 2.8, 2.0 Hz, Ar), 5.13 (s, 2 H, CH_2Ph), 3.46 (t, 2 H, *J* = 6.3 Hz, CH_2), 3.35 (t, 2 H, *J* = 6.3 Hz, CH_2).

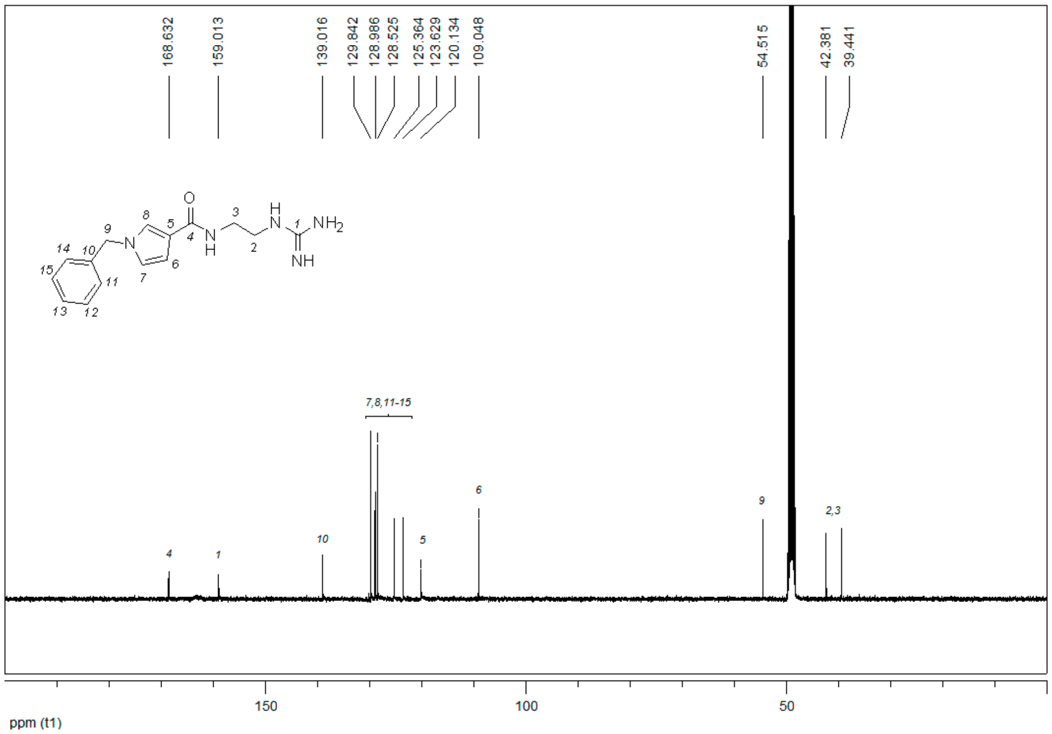

Figure S4. ¹³C NMR spectra of analogue **15** (100 MHz, CD_3OD) δ 168.6 (C=O), 159.0 (C=NH), 139.0 (C Ph), 129.8 (2 × CH Ph), 129.0 (CH), 128.5 (2 × CH Ph), 125.4 (CH), 123.6 (CH), 120.1 (C Ar), 109.0 (CH Ar), 54.5 (CH_2Ph), 42.4 (CH_2), 39.4 (CH_2).

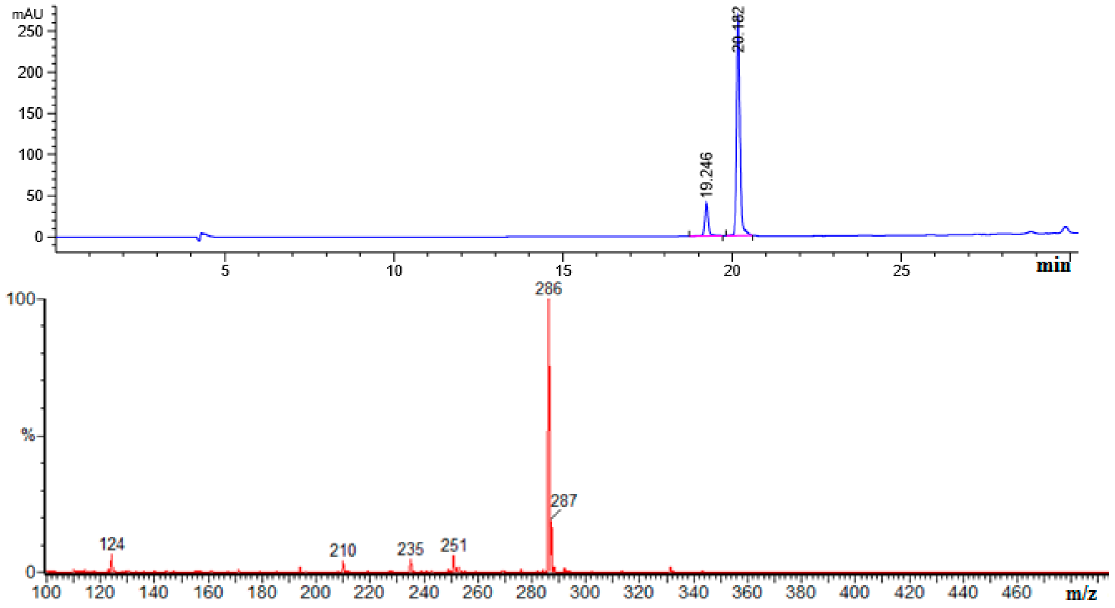

Figure S5. RP-HPLC chromatogram (top) and ESI-MS (bottom) of final analogue **16** ($MW_{theoretical}$: 285.34).

RP-HPLC Conditions:

i) Column: Agilent ZORBAX Eclipse Plus C18 (3.5μm, 100x4.6mm),

ii) Solvents: H_2O (0.08% TFA), AcN (0.08%TFA),

iii) Gradient elution: from 5% AcN to 100% AcN over 30min.

iv) t_R: 20.2 min.

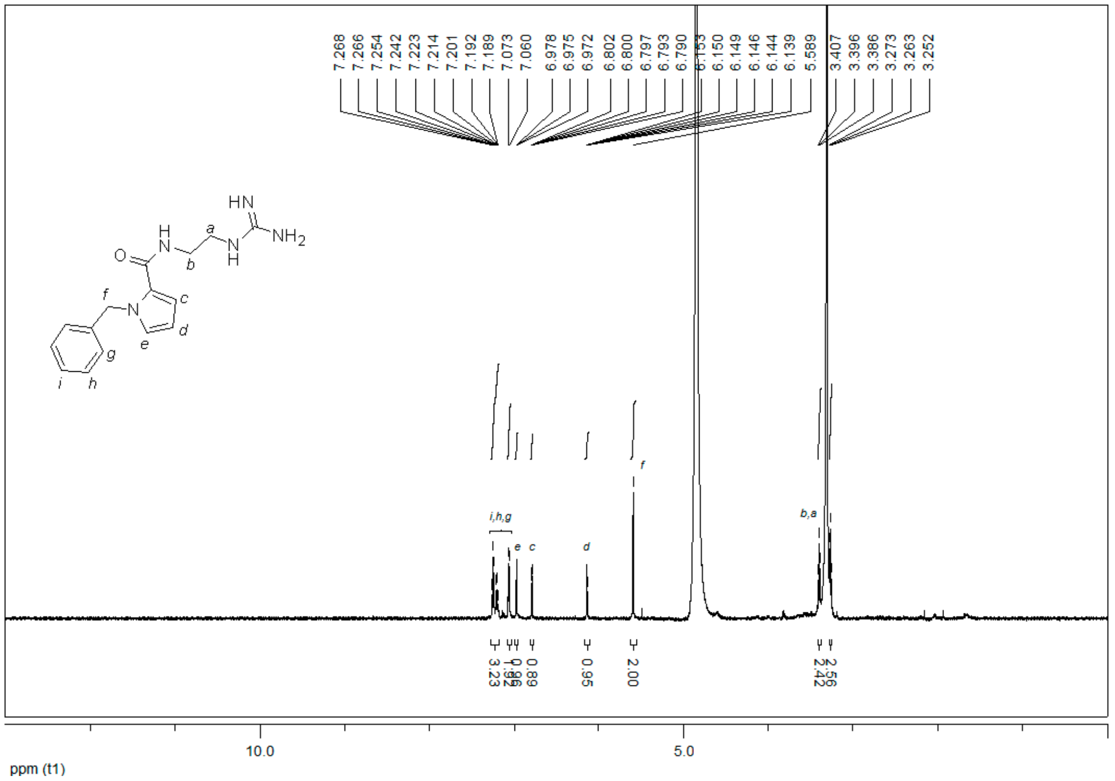

Figure S6. ^{1}H NMR spectra of analogue **16** (600 MHz, CD_3OD) δ 7.19-7.27 (m, 3 H, Ph), 7.07 (d, 2 H, = 7.8 Hz, Ph), 6.97-6.98 (m, 1 H, Ar), 6.79-6.80 (m, 1 H, Ar), 6.14-6.15 (m, 1 H, Ar), 5.59 (s, 2 H, 3.40 (t, 2 H, *J* = 6.3 Hz, CH_2), 3.26 (t, 2 H, *J* = 6.3 Hz, CH_2).

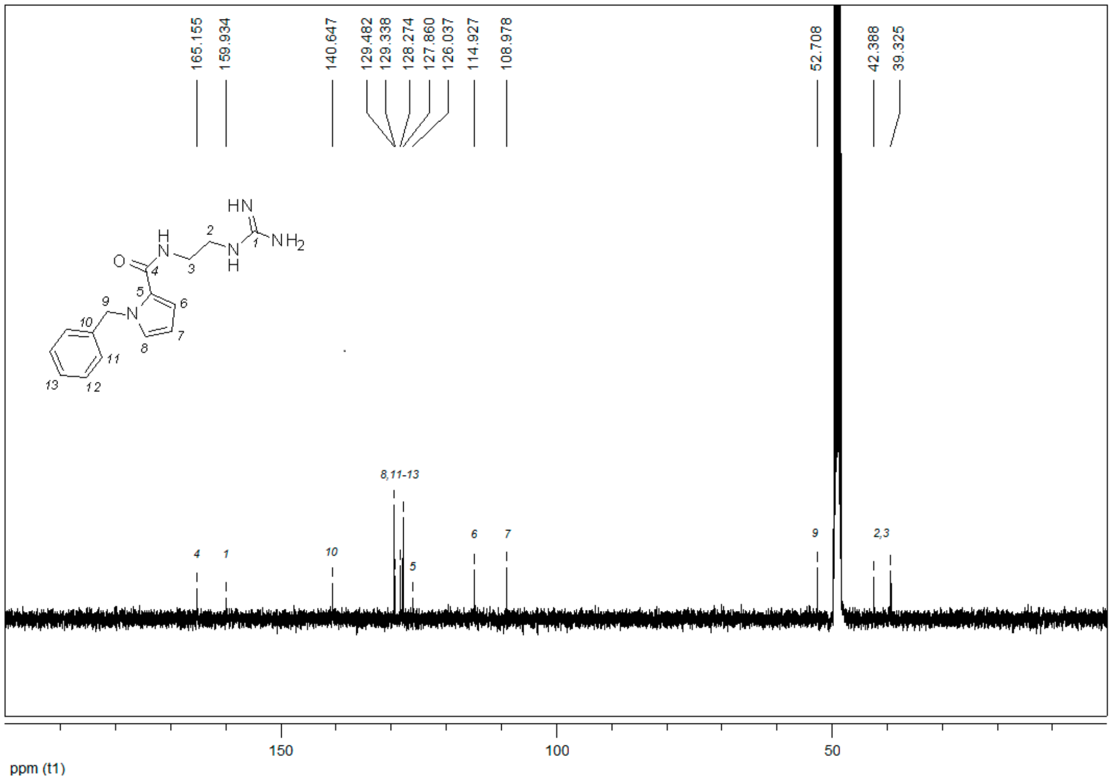

Figure S7. ^{13}C NMR spectra of analogue **16** (100 MHz, CD_3OD) δ 165.2 (C=O), 159.9 (C=NH), 140.6 Ph), 129.5 (2 × CH Ph), 129.3 (CH), 128.3 (CH), 127.9 (2 × CH Ph), 126.0 (C Ar), 114.9 (CH Ar), 109.0 (CH Ar), 52.7 (CH_2Ph), 42.4 (CH_2), 39.3 (CH_2).

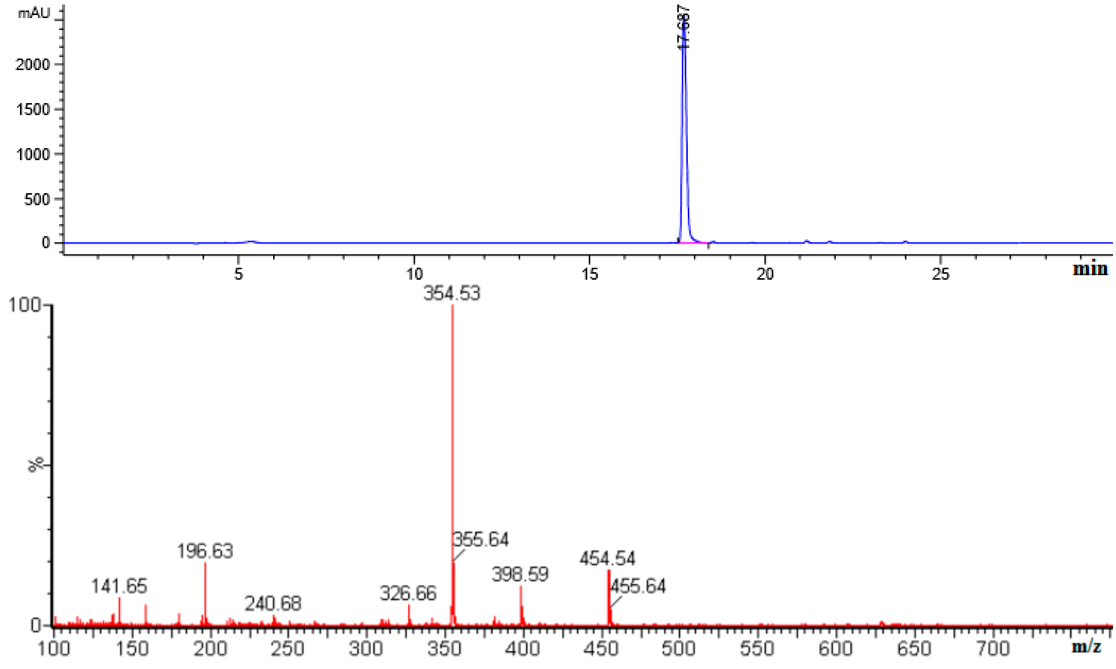

Figure S8. RP-HPLC chromatogram (top) and ESI-MS (bottom) of final analogue **17** ($MW_{theoretical}$: 353.48).

RP-HPLC Conditions:

i) Column: Agilent ZORBAX Eclipse Plus C18 (3.5μm, 100x4.6mm),

ii) Solvents: H_2O (0.08% TFA), AcN (0.08%TFA),

iii) Gradient elution: from 10% AcN to 100% AcN over 30min.

iv) t_R: 17.7 min, Purity: 99%

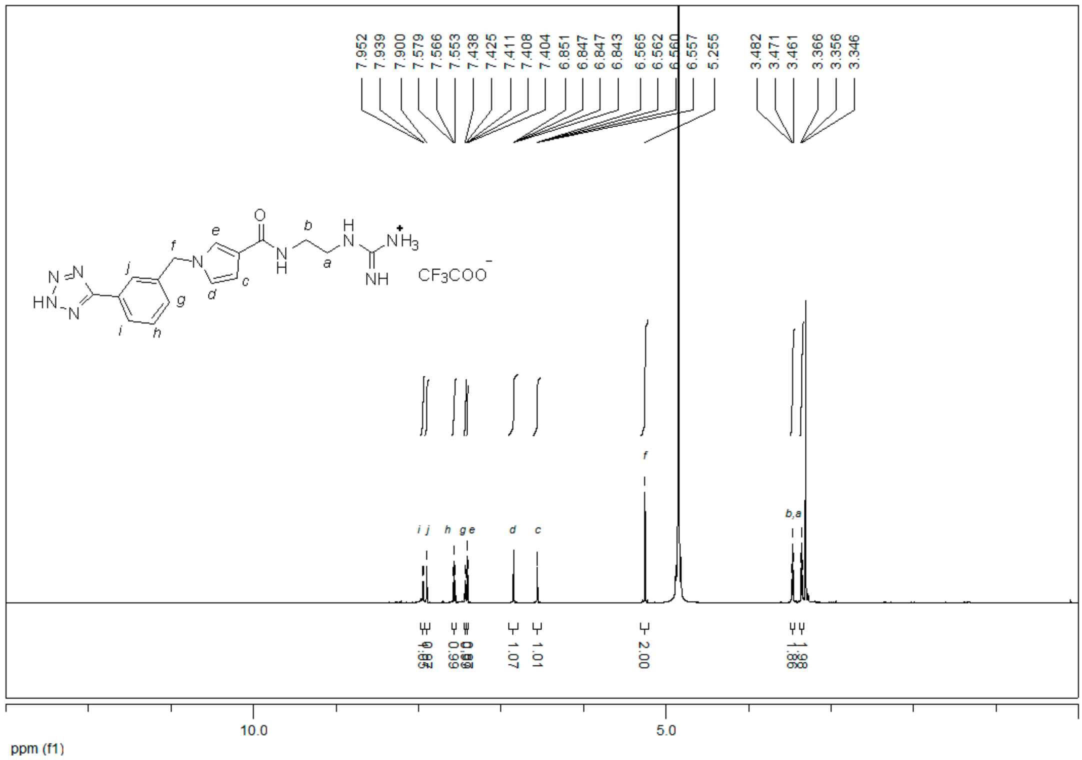

Figure S9. ^{1}H NMR spectra of analogue **17** (600 MHz, CD_3OD) δ 7.95 (d, 1 H, *J* = 7.8 Hz, Ar′), 7.90 (s, 1 H, Ar′), 7.57 (t, 1 H, *J* = 7.8 Hz, Ar′), 7.43 (d, 1 H, *J* = 7.8 Hz, Ar′), 7.41 (app dd, 1 H, *J* = 2.4, 1.8 Hz, Ar), 6.85 (dd, 1 H, *J* = 3.0, 2.4 Hz, Ar), 6.56 (dd, 1 H, *J* = 3.0, 1.8 Hz, Ar), 5.26 (s, 2 H, CH_2Ar′), 3.47 (t, 2 H, *J* = 6.0 Hz, CH_2), 3.36 (t, 2 H, *J* = 6.0 Hz, CH_2).

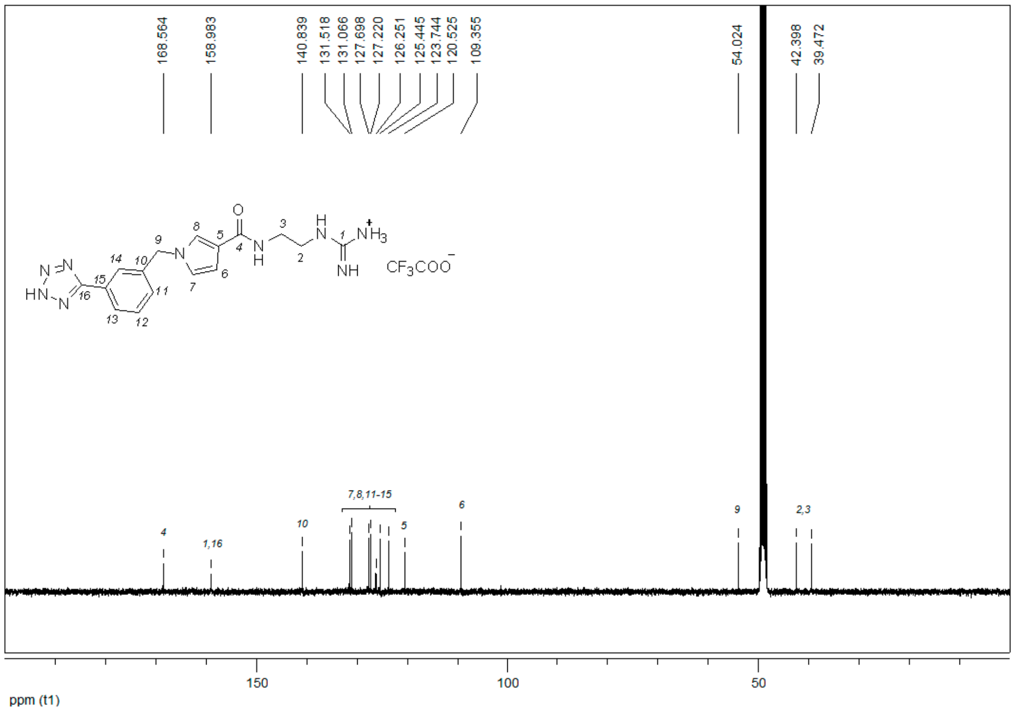

Figure S10. ^{13}C NMR spectra of analogue **17** (100 MHz, CD_3OD) δ 168.6 (C=O), 159.0 (2 × C=NH), 140.8 (C Ar′), 131.5 (CH), 131.1 (CH), 127.7 (CH), 127.2 (CH), 126.3 (C Ar′), 125.4 (CH), 123.7 (CH), 120.5 (C Ar), 109.4 (CH Ar), 54.0 (CH_2Ar′), 42.4 (CH_2), 39.5 (CH_2).

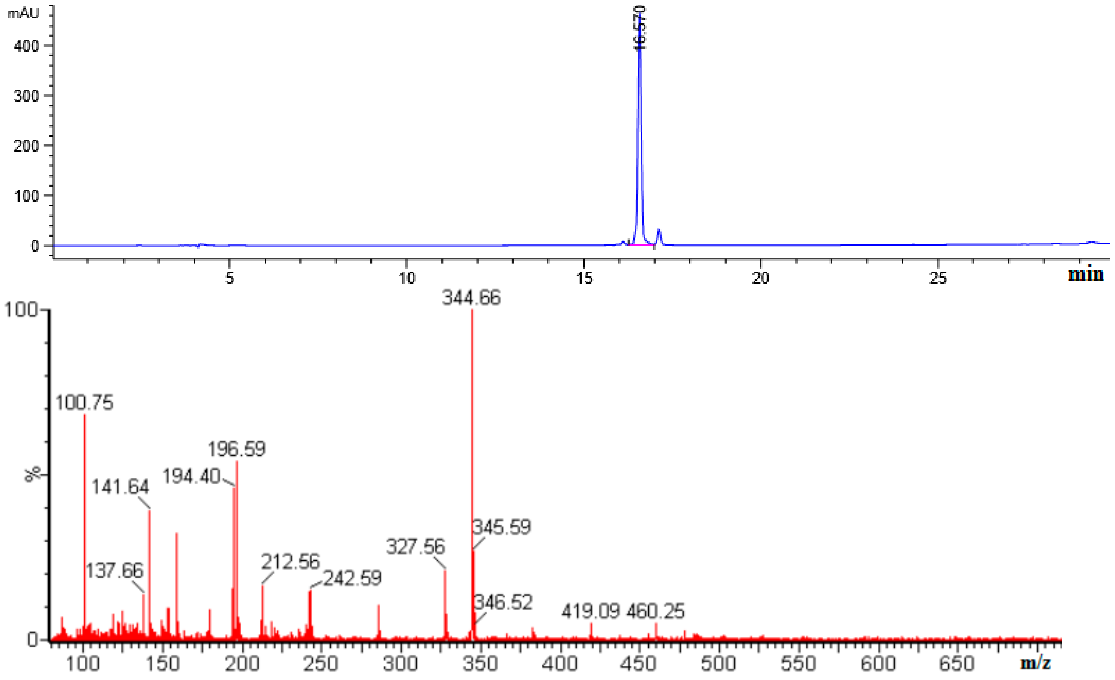

Figure S11. RP-HPLC chromatogram (top) and ESI-MS (bottom) of final analogue **18** ($MW_{theoretical}$: 343.39).

RP-HPLC Conditions:

i) Column: Agilent ZORBAX Eclipse Plus C18 (3.5μm, 100x4.6mm),

ii) Solvents: H_2O (0.08% TFA), AcN (0.08%TFA),

iii) Gradient elution: from 10% AcN to 100% AcN over 30min.

iv) t_R: 16.6 min, Purity: 98%.

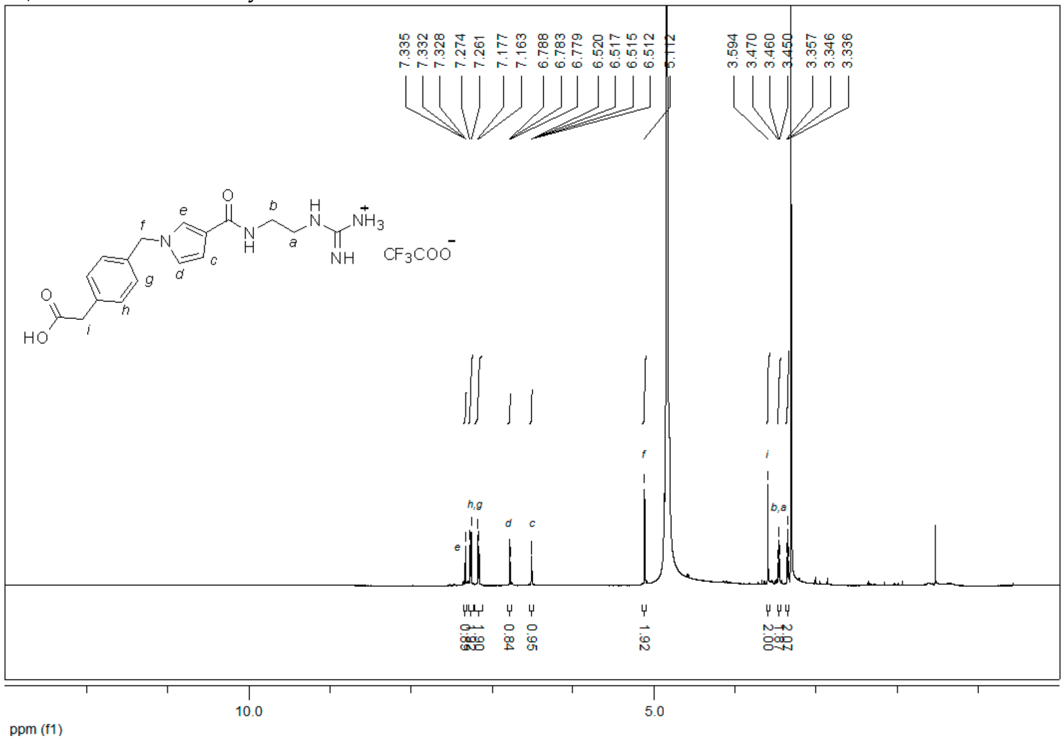

Figure S12. ^{1}H NMR spectra of analogue **18** (600 MHz, CD_3OD) δ 7.33 (app dd, 1 H, *J* = 2.4, 1.8 Hz, Ar), 7.27 (d, 2 H, *J* = 8.1 Hz, Ar’), 7.17 (d, 2 H, *J* = 8.1 Hz, Ar’), 6.78 (dd, 1 H, *J* = 3.0, 2.4 Hz, Ar), 6.52 (dd, 1 H, *J* = 3.0, 1.8 Hz, Ar), 5.11 (s, 2 H, CH_2Ar'), 3.59 (s, 2 H, CH_2CO_2H), 3.46 (t, 2 H, *J* = 6.0 Hz, CH_2), 3.35 (t, 2 H, *J* = 6.0 Hz, CH_2).

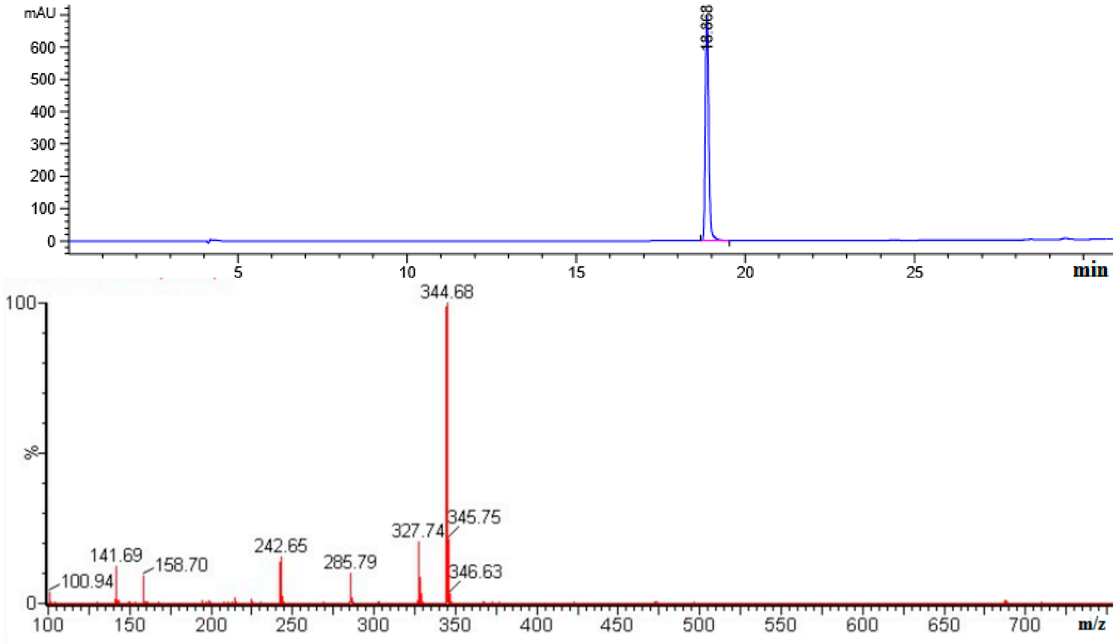

Figure S13. RP-HPLC chromatogram (top) and ESI-MS (bottom) of final analogue **19** ($MW_{theoretical}$: 343.38).
RP-HPLC Conditions:
i) Column: Agilent ZORBAX Eclipse Plus C18 (3.5µm, 100x4.6mm),
ii) Solvents: H_2O (0.08% TFA), AcN (0.08%TFA),
iii) Gradient elution: from 5% AcN to 100% AcN over 30min.
iv) t_R: 18.9 min, Purity: 99%.

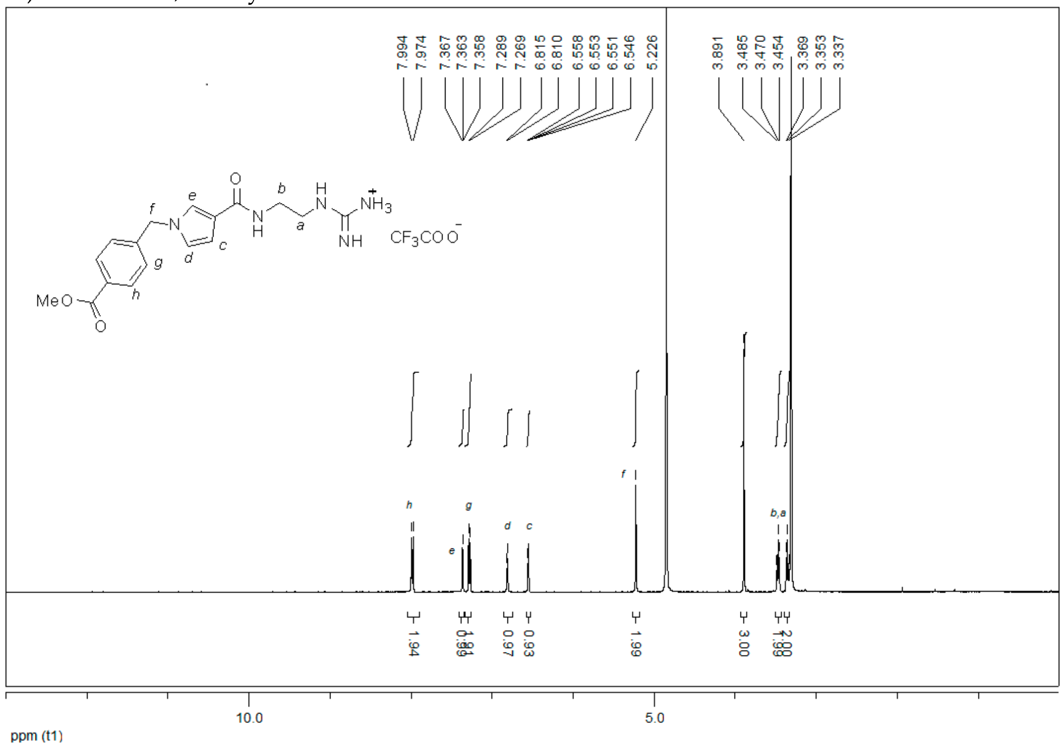

Figure S14. ^{1}H NMR spectra of analogue **19** (400 MHz, CD_3OD) δ 7.98 (d, 2 H, *J* = 8.0 Hz, Ar'), 7.36 (app t, 1 H, *J* = 2.0 Hz, Ar), 7.28 (d, 2 H, *J* = 8.0 Hz, Ar'), 6.81-6.82 (m, 1 H, Ar), 6.55 (dd, 1 H, *J* = 2.8, 2.0 Hz, Ar), 5.23 (s, 2 H, CH_2Ar'), 3.89 (s, 3 H, OCH_3), 3.47 (t, 2 H, *J* = 6.4 Hz, CH_2), 3.35 (t, 2 H, *J* = 6.4 Hz, CH_2).
